# Dynamic perturbation analysis: a modular rewiring framework for decoding cancer stage progression and identifying therapeutic vulnerabilities

**DOI:** 10.1093/bib/bbag572

**Published:** 2026-10-19

**Authors:** Bin Yang, Mi Li, Xingyi Liu, Xiaojie Jin, Guang Hu

**Affiliations:** Biomedical Basic Research Center (BBRC) of Jiangsu, MOE Key Laboratory of Geriatric Diseases and Immunology, School of Life Sciences, Soochow University, No. 199, Ren'ai Road, Suzhou Industrial Park, Suzhou, Jiangsu, 215123, China; College of Pharmacy, Gansu University of Chinese Medicine, No. 35, Dingxi East Road, Chengguan District, Lanzhou, Gansu, 730000, China; Biomedical Basic Research Center (BBRC) of Jiangsu, MOE Key Laboratory of Geriatric Diseases and Immunology, School of Life Sciences, Soochow University, No. 199, Ren'ai Road, Suzhou Industrial Park, Suzhou, Jiangsu, 215123, China; College of Pharmacy, Gansu University of Chinese Medicine, No. 35, Dingxi East Road, Chengguan District, Lanzhou, Gansu, 730000, China; Biomedical Basic Research Center (BBRC) of Jiangsu, MOE Key Laboratory of Geriatric Diseases and Immunology, School of Life Sciences, Soochow University, No. 199, Ren'ai Road, Suzhou Industrial Park, Suzhou, Jiangsu, 215123, China; Institute of Health Data Science at Soochow University, No. 199, Ren'ai Road, Suzhou Industrial Park, Suzhou, Jiangsu, 215123, China

**Keywords:** dynamic perturbation analysis, modular rewiring, disease progression

## Abstract

Disease progression is driven not only by transcriptional alterations but also by dynamic reorganization of molecular interaction networks. However, most existing approaches focus on differential gene expression and fail to capture modular-level reorganization. Here, we present dynamic perturbation analysis (DPA), a modular rewiring framework that quantifies dynamic perturbations across disease stages using a novel metric termed the degree of perturbation. Using gastric cancer (GC) progression as a model system, DPA revealed that extensive modular rewiring persists despite limited stage-specific transcriptional changes after tumor initiation. DPA identified progression-associated perturbation programs characterized by extracellular matrix remodeling, oncogenic signaling activation, stemness acquisition, and late-stage microenvironmental adaptation. Perturbation analysis revealed two major disease-driving programs and prioritized previously unrecognized regulators, including protein tyrosine phosphatase non-receptor type 12 (PTPN12) and leucine rich repeat and Ig domain containing 1 (LINGO1), whose functional roles in GC progression were experimentally validated. Furthermore, modular rewiring-guided drug repurposing uncovered PJ-34 as a promising therapeutic candidate targeting late-stage disease-associated perturbations. Collectively, DPA provides a generalizable framework for decoding disease progression through dynamic modular rewiring, enabling the identification of driver programs, candidate regulators, and therapeutic vulnerabilities. This framework offers a scalable strategy for network-guided mechanistic discovery and therapeutic prioritization in complex diseases.

## Graphical Abstract

**Graphical Abstract:**
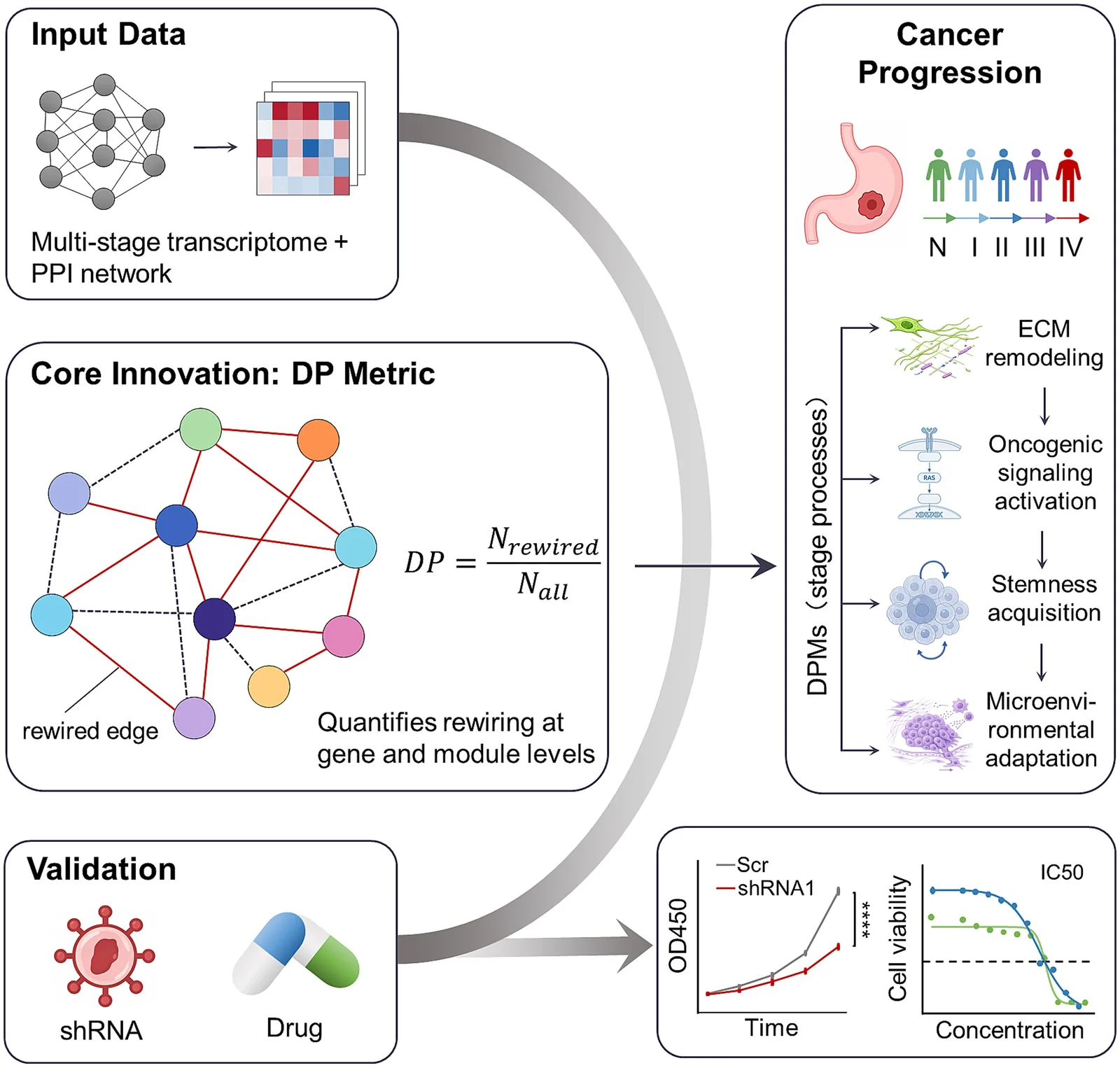

## Introduction

Cancer progression is a dynamic evolutionary process characterized by the continuous acquisition of malignant phenotypes and the progressive remodeling of cellular regulatory systems [1]. Emerging evidence indicates that tumor progression is driven not solely by genomic alterations but also by adaptive transitions among distinct cellular states, accompanied by extensive reorganization of signaling pathways, metabolic programs, and tumor–microenvironment interactions [2, 3]. These dynamic transitions underlie tumor plasticity, therapeutic resistance, and metastatic dissemination [4], underscoring the need for systems-level approaches capable of capturing disease progression beyond static molecular snapshots.

Transcriptomic profiling has substantially advanced our understanding of cancer biology and enabled the discovery of numerous disease-associated genes and pathways [5]. However, accumulating evidence suggests that transcriptional alterations alone often fail to fully account for the phenotypic transitions observed during cancer progression [6–8]. In many cancers, including gastric cancer (GC) [9], substantial changes in invasion, stemness, immune remodeling, and therapeutic response can occur despite relatively modest transcriptional differences between adjacent disease stages. These observations suggest that disease stage transitions are associated not only with changes in gene abundance but also with dynamic reorganization of molecular interaction networks [10, 11].

Network medicine has emerged as a powerful framework for understanding complex diseases from a systems perspective [12–14]. By integrating molecular interaction networks with multiomics data, network-based approaches have successfully identified disease modules, prioritized therapeutic targets, and revealed mechanistic relationships linking molecular perturbations to clinical phenotypes [15–17]. More recently, network medicine has evolved from static disease–gene association studies toward the characterization of disease states and dynamic network perturbations [18]. Emerging computational frameworks have increasingly focused on modeling disease progression through dynamic network analysis. For example, CancerHubs identified disease-associated hub dynamics across cancer networks [19], DyNDG characterized dynamic network dependency relationships underlying disease progression [20], DiCE systematically captured context-specific interaction rewiring events [21], and TransMarker leveraged temporal network information for disease biomarker discovery [22]. In particular, growing evidence suggests that disease progression is accompanied by extensive network-state remodeling, whereby alterations in molecular interactions reshape cellular regulatory programs and drive phenotypic transitions [23]. Such interaction-level remodeling may provide mechanistic information that is not captured by conventional differential expression analyses.

Among existing network-based approaches, module-level analyses have proven especially effective for identifying coordinated biological processes underlying disease development [24–29]. Recent studies have demonstrated the utility of network modules in characterizing tumor ecosystems, identifying disease-associated pathways, revealing signaling transitions, and predicting therapeutic responses. In particular, oncogenic allosteric modules revealed how allosteric communication networks coordinate cancer-associated signaling programs [30], ProgModule identified progression-associated functional modules across disease stages [31], FunMap systematically reconstructed functional interaction modules from large-scale transcriptomic data [32], and genome-scale functional module (GSFM) integrated gene-set and functional-module information to uncover disease-relevant regulatory programs [33]. Building upon this concept, our previous studies developed a series of network module-based approaches for disease mechanism discovery and therapeutic prioritization. We established computational frameworks for identifying disease-associated functional modules and quantifying network perturbations across biological systems [34, 35], and further demonstrated their utility in systems-level drug discovery, drug repositioning, and therapeutic target identification across multiple disease contexts [36–38]. These studies highlighted the potential of network modules as mechanistic units linking molecular perturbations to disease phenotypes and therapeutic responses. Nevertheless, several important challenges remain unresolved. First, current approaches mainly focus on identifying differential interactions, network hubs, or role-transition genes, while lacking a unified framework for quantitatively measuring the extent of network perturbation during disease progression. Second, the relationships among gene-level, edge-level, and module-level perturbations remain poorly characterized. Third, existing dynamic network approaches rarely connect network-state remodeling with therapeutic opportunities, limiting their translational value.

To address these challenges, we developed dynamic perturbation analysis (DPA), a modular rewiring framework for quantifying disease-state transitions and therapeutic vulnerabilities. Using GC as a model system, DPA integrated stage-specific transcriptomic profiles with protein–protein interaction (PPI) networks to reconstruct progression-associated network patterns. The framework identified extensive interaction rewiring, persistent disease-driving programs, candidate regulators, and actionable therapeutic vulnerabilities. More broadly, DPA extends network medicine from static disease characterization toward quantitative modeling of disease progression and provides a scalable strategy for linking network dynamics to mechanistic discovery and therapeutic prioritization.

## Materials and methods

In this section, we introduce the framework of DPA. Detailed descriptions of conventional bioinformatics analyses and experimental procedures are provided in the Supporting Information.

### Overview of DPA

DPA was developed as an extension of our previously established network module and perturbation analysis frameworks, which demonstrated that disease-associated biological processes are frequently reflected by alterations in network modular organization rather than individual gene expression changes [34]. DPA extends conventional differential network and module analyses by explicitly quantifying interaction-level perturbations across sequential disease states and reconstructing progression-associated rewiring patterns. The DPA workflow consists of three major steps (Fig. 1) as follows: (i) construction of stage-specific dynamic interactomes using transcriptomic profiles and a reference PPI network, followed by the identification of network modules within each disease state; (ii) module-guided detection of interaction rewiring during stage transitions; and (iii) quantification of dynamic perturbations at gene, interaction, and module levels using the degree of perturbation (DP). Finally, dynamic perturbation modules (DPMs), stage-associated driver genes, and therapeutic vulnerabilities were identified from progression-associated rewiring patterns.

**Figure 1:**
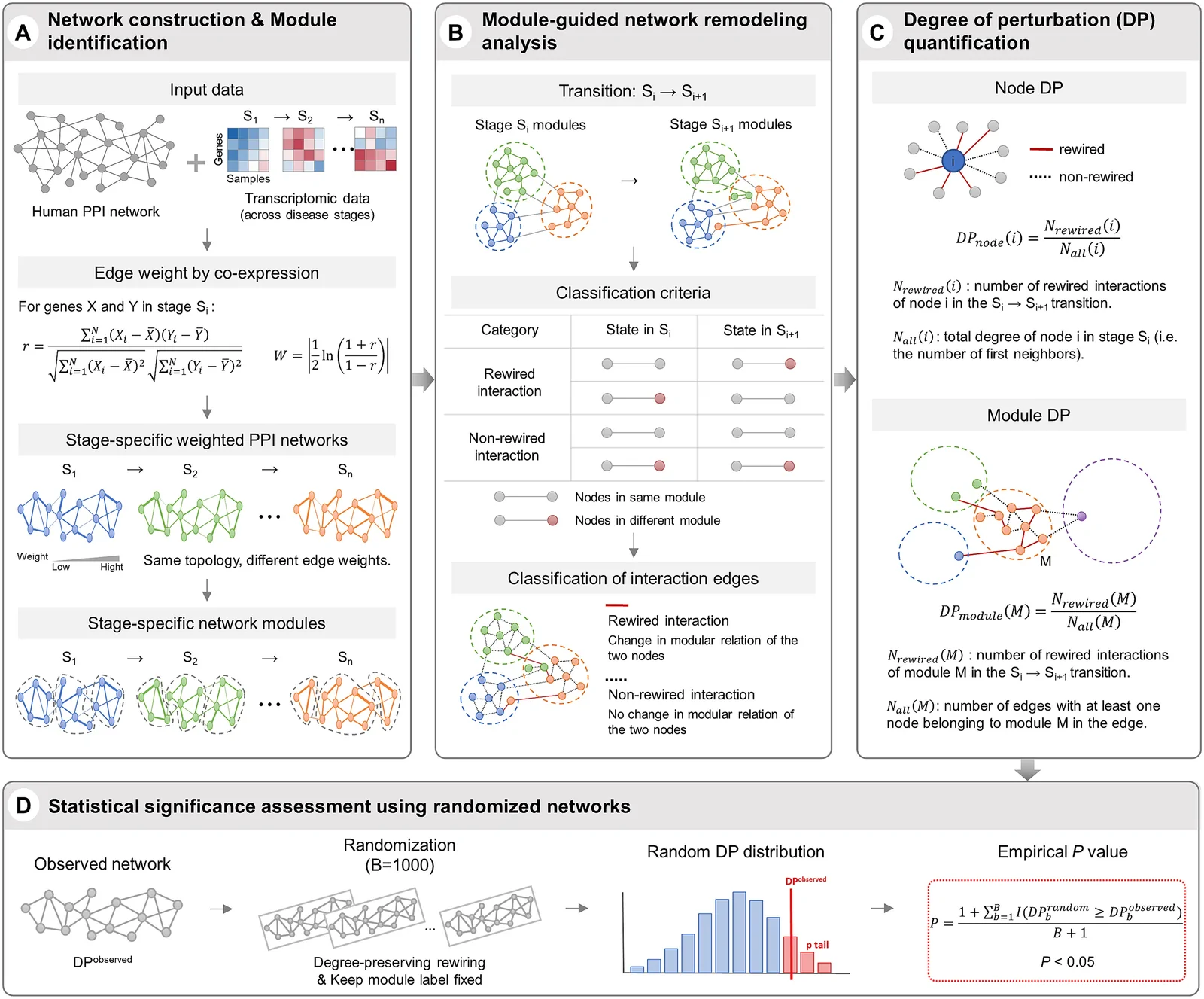
Pipeline of the DPA framework. (A) Transcriptomic profiles from different disease stages were integrated with the human interactome to construct dynamic stage-specific interactomes, from which network modules were subsequently identified to characterize the modular organization of each disease stage. (B) Changes in modular relationships between adjacent disease stages were compared to perform modular rewiring analysis, thereby identifying rewired interactions whose modular relationships changed and nonrewired interactions whose modular relationships remained unchanged. (C) Based on modular rewiring events, the DP was calculated at both the node and module levels to quantify dynamic network perturbations during disease progression. (D) The statistical significance assessment of DPA.

### Dynamic weighted network construction and module identification

To characterize dynamic changes in interaction patterns during disease progression, we constructed dynamic stage-specific interactomes for each disease stage (Fig. 1A). First, genes with reliable expression across all disease stages were retained following expression filtering. Human protein interactome data were then obtained from the Search Tool for the Retrieval of Interacting Genes/Proteins (STRING) database [39], and only interactions with a confidence score ≥ 400 were retained. The filtered genes were mapped onto the human interactome to establish a common PPI network backbone.

Subsequently, transcriptomic profiles from samples belonging to each disease stage (*S_i_*) were used to dynamically assign weights to the network, thereby generating dynamic state-specific interactomes. For any interacting genes *X* and *Y*, the Pearson correlation coefficient (*r*) was first calculated as follows:


(1) \begin{eqnarray*} r=\frac{\sum_{i=1}^N\left({X}_i-\overline{X}\right)\left({Y}_i-\overline{Y}\right)}{\sqrt{\sum_{i=1}^N{\left({X}_i-\overline{X}\right)}^2}\sqrt{\sum_{i=1}^N{\left({Y}_i-\overline{Y}\right)}^2}}, \end{eqnarray*}

where *N* represents the number of samples in stage *S_i_*, ${X}_i$ and ${Y}_i$ denote the expression values of genes *X* and *Y* in the *i*th sample, respectively; and $\overline{X}$ and $\overline{Y}$ represent the mean expression levels of genes *X* and *Y* across all samples. The interaction weights are then defined as the absolute value of the Pearson correlation coefficients transformed using Fisher’s *Z*-transformation [40] as follows:


(2) \begin{eqnarray*} W=\left|\frac{1}{2}\ln \left(\frac{1+r}{1-r}\right)\right| \end{eqnarray*}

By repeating this process across all disease progression stages (*S*_1_, *S*_2_,…, *S_n_*). A series of dynamic stage-specific interactomes were generated by integrating transcriptomic profiles from individual disease stages with the common PPI backbone. These networks shared an identical topological structure, whereas the edge weights were determined by stage-specific gene coexpression patterns, enabling the characterization of dynamic interaction rewiring during disease progression.

Based on the constructed dynamic stage-specific interactomes, we performed network module detection for each disease stage to characterize the modular organization of the network (Fig. 1A). Weighted network modules were identified using the Fast Unfolding Louvain algorithm based on modularity optimization [41]. This algorithm iteratively maximizes network modularity to partition the network into densely connected modules with relatively sparse rewired interactions, thereby uncovering the hierarchical organization of the network. Specifically, the algorithm consists of two alternating steps: (i) local optimization, in which each node is initially treated as an independent community, and nodes are iteratively reassigned to neighboring communities according to the resulting changes in modularity, with each node being assigned to the community that maximizes the modularity gain and (ii) community aggregation, in which nodes belonging to the same community are merged into a super-node once community assignments become stable, thereby generating a compressed network on which the local optimization process is repeated. These two steps are iteratively performed until no further increase in network modularity can be achieved, resulting in a stable module partition. To ensure the reproducibility and comparability of module detection across disease states, the random seed was set to 1 and the resolution parameter was set to 2.

### Module-guided network remodeling analysis

Although PPI networks from different stages shared the same topological structure, differences in edge weights resulted in distinct module partitions across stages. We hypothesized that alterations in module membership reflect large-scale reorganization of modular architecture and therefore serve as indicators of network-state transitions during disease progression. To quantify perturbed PPIs undergoing modular reorganization during disease progression, we developed a module-guided network remodeling strategy (Fig. 1B).

Taking the transition between two adjacent disease stages, *S_i_* and *S*_*i* + 1_, as an example, each interaction was categorized according to the modular relationship of its two interacting nodes: (i) the two nodes were assigned to the same module or (ii) the two nodes were assigned to different modules. As the disease state progressed from *S_i_* to *S*_*i* + 1_, the module relationship between interacting nodes could change, resulting in either stable or altered modular relationships for individual interactions. All interactions during the *S_i_* → *S*_*i* + 1_ transition were classified into two categories: rewired interactions and nonrewired interactions (Table 1). A nonrewired interaction represents an interaction whose modular relationship remains unchanged between *S_i_* and *S*_*i* + 1_, meaning that two interacting nodes consistently belonged either to the same module or to different modules across both stages. In contrast, a rewired interaction represents an interaction whose modular relationship has changed during the stage transition, indicating that the two nodes belonged to the same module in *S_i_* but were assigned to different modules in *S*_*i* + 1_, or vice versa.

**Table 1:** Classification criteria of interactions during disease progression.

Category	Module membership of interacting nodes in *S*_*i*_	Module membership of interacting nodes in *S*_*i* + 1_
Rewired interaction	Same module	Different modules
	Different modules	Same module
Nonrewired interaction	Same module	Same module
	Different module	Different module

Accordingly, the PPI topology remains identical across all stages, and this classification reflects changes in module assignments driven by stage-specific coexpression patterns, rather than physical gain or loss of PPI. We therefore use the term “modular rewiring” throughout to emphasize that the observed reorganization occurs at the module level within a fixed interaction backbone.

### DP quantification

Rewired interactions not only intuitively reflect the reorganization of modular architecture during disease progression but have also been shown to represent critical changes in the interaction relationships that drive disease progression, inducing system level network disturbances and facilitating the identification of key driver genes and potential therapeutic targets [34]. Based on this concept, the DP metric was designed to quantify the extent to which a node participates in disease-associated network-state transitions. Rather than measuring expression changes, DP captures interaction-level perturbations arising from rewiring events within the molecular network (Fig. 1C).

For a given node *i*, the DP value was defined as the proportion of rewired interactions associated with that node as follows:


(3) \begin{eqnarray*} {\mathrm{DP}}_{\mathrm{node}}(i)=\frac{N_{\mathrm{rewired}}(i)}{N_{\mathrm{all}}(i)} \end{eqnarray*}

where ${N}_{\mathrm{all}}(i)$ represents the total number of associated interactions for node *i*, defined as all interactions between node *i* and its first-order neighbors, and ${N}_{\mathrm{rewired}}(i)$ denotes the number of those interactions classified as rewired interactions. A high DP value indicates that the node undergoes extensive interaction rewiring during disease progression, whereas a low DP value suggests that its interaction patterns remain relatively stable.

To evaluate the overall perturbation of functional modules, DP was further extended to the module level. For a given module *M*, the DP value was defined as the proportion of rewired interactions associated with that module:


(4) \begin{eqnarray*} {\mathrm{DP}}_{\mathrm{module}}(M)=\frac{N_{\mathrm{rewired}}(M)}{N_{a\mathrm{ll}}(M)} \end{eqnarray*}

where ${N}_{\mathrm{all}}(M)$ represents the total number of interactions associated with module *M*, defined as interactions involving at least one member node of the module, and ${N}_{\mathrm{rewired}}(M)$ represents the subset of these interactions classified as rewired interactions. Module-level DP reflects the extent of structural reorganization experienced by a module during disease progression. A higher DP value indicates more extensive modular rewiring and the greater the perturbation it experiences. To characterize the temporal evolution of network perturbations, DP values calculated across sequential disease transitions were assembled into perturbation trajectories. Modules exhibiting similar DP dynamics were grouped using hierarchical clustering, enabling the identification of persistent and stage-specific perturbation programs. To further evaluate the robustness and biological specificity of DPA, additional analyses including statistical significance assessment (Fig. 1D), resampling, covariate adjustment, and benchmark evaluation were performed.

## Results

### DPA reveals extensive modular rewiring beyond transcriptomic alterations during GC progression

To explore the molecular alterations underlying GC, we first evaluated the transcriptomic characteristics across different disease stages. Principal component analysis revealed a clear separation between normal gastric tissues and tumor samples, whereas samples from different tumor stages largely overlapped and failed to form distinct stage-specific clusters (Supplementary Fig. S1A). Accordingly, differential expression analysis identified 3533 differential expressed genes (DEGs) between normal tissue and stage I tumors, but only 134, 10, and 77 DEGs during T1 → T2,T2 → T3, and T3 → T4 transitions, respectively (Supplementary Fig. S1B). These findings indicate that while transcriptomic alterations are most pronounced during tumor initiation, inter-stage expression differences are comparatively modest, suggesting that conventional expression-based approaches may be insufficient to fully capture the molecular reorganization driving cancer progression [42–44].

To investigate whether disease progression is accompanied by network-level remodeling beyond gene expression changes, we applied the DPA framework to reconstruct dynamic weighted PPI networks for normal tissues and four GC stages. By integrating transcriptomic profiles with the human interactome (Supplementary Fig. S1C), we generated five stage-specific weighted PPI networks and identified 25, 30, 26, 26, and 24 network modules in the N, T1, T2, T3, and T4 states, respectively (Fig. 2A). These modules represent the fundamental functional units through which network reorganization can be systematically characterized during disease progression.

**Figure 2:**
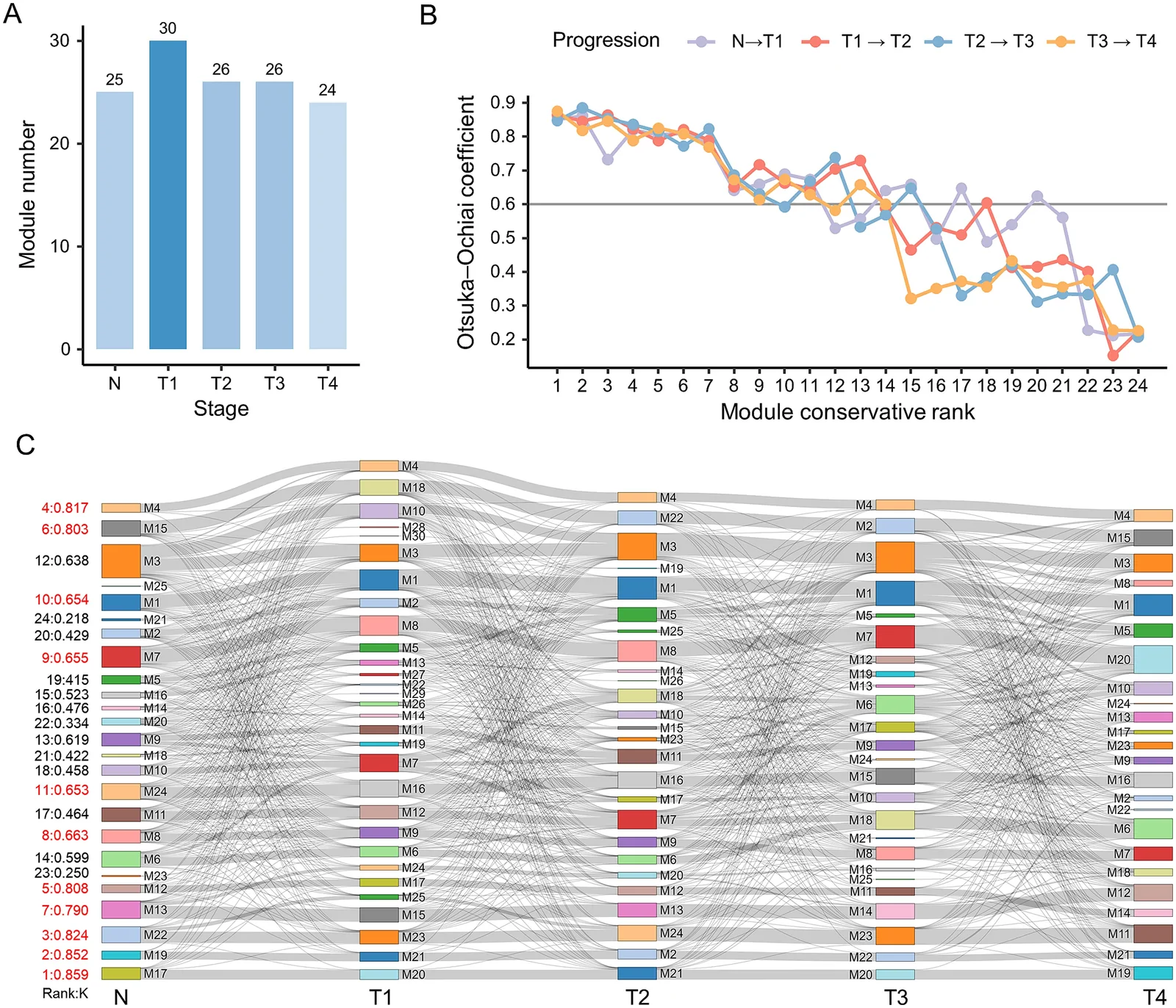
Module conservation analysis across GC progression stages. (A) Overview of module identification in weighted PPI networks for N, T1, T2, T3, and T4 stages. (B) Conservation scores (maximum *K* values) of *N*-group modules across subsequent progression processes. The *x*-axis represents the rank of the average *K* value for each module, arranged in ascending order. (C) Correspondence relationships of modules across GC progressions. The average *K* value of the conserved module marked in red (i.e. the average value of the main corresponding module from N to T4). See also Supplementary Table S1.

We next evaluated module conservation across consecutive disease stages using the Otsuka–Ochiai coefficient (*K*). Several modules exhibited high conservation throughout progression, with *K* values frequently exceeding 0.6 across multiple stage transitions (Fig. 2B). Based on these module correspondence patterns, 11 modules were classified as conserved modules, whereas the remaining modules were designated perturbed modules. Functional enrichment analysis revealed that conserved modules were primarily involved in essential cellular processes, including ribosome biogenesis, cell-cycle regulation, oxidative phosphorylation, protein homeostasis, and endocytosis (Table 2 and Supplementary Fig. S2A). These findings suggest that core cellular functions remain largely preserved throughout GC progression.

**Table 2:** Functional categorization of module functions.

Functional category	Conserved modules	Perturbed modules
Genetic information processing and proliferation	Ribosome Ribosome biogenesis in eukaryotes Spliceosome Cell cycle ATP-dependent chromatin remodeling	——
Energy and metabolic processes	Oxidative phosphorylation	Glycerophospholipid metabolism Sphingolipid metabolism Inositol phosphate metabolism
Protein homeostasis and transport	Endocytosis Ubiquitin-mediated proteolysis	Protein processing in endoplasmic reticulum Autophagy—animal
Cellular structure and motility	Motor proteins	Integrin signaling Cadherin signaling Cytoskeleton in muscle cells Cornified envelope formation
Signal transduction and environmental interaction	Adrenergic signaling in cardiomyocytes Cytokine–cytokine receptor interaction	Neuroactive ligand–receptor interaction Dopaminergic synapse Hippo signaling pathway Circadian rhythm

In contrast, perturbed modules were predominantly enriched in pathways associated with environmental adaptation, intercellular communication, signaling transduction, and metabolic plasticity (Table 2 and Supplementary Fig. S2B). Notably, tracing module evolutionary relationships across stages revealed that several modules with relatively low conservation scores still retained a substantial proportion of their original member genes (Fig. 2C and Supplementary Table S1). For example, modules M21, M23, and M11 maintained extensive node overlap with their descendant modules despite exhibiting low *K* values. This observation suggests that module composition alone cannot fully capture the dynamic reorganization occurring during disease progression. Instead, substantial rewiring of interaction relationships may occur even when module membership remains largely unchanged.

Collectively, these results reveal a striking discrepancy between transcriptomic stability and network dynamics during GC progression. Although relatively few genes exhibit stage-specific expression changes after tumor initiation, extensive reorganization of interaction patterns persists across disease stages. Moreover, module conservation based solely on node composition fails to adequately quantify such dynamic remodeling. These findings provide the rationale for developing a rewiring-based perturbation metric capable of directly measuring modular-level reorganization during disease progression.

### DP captures biologically meaningful network perturbation during GC progression

The DPA framework identifies modular rewiring events by comparing changes in modular relationships across disease stages and introduces a new metric, termed the DP, to quantitatively assess the extent of network perturbation experienced by nodes and modules during disease progression (Fig. 3A). To systematically benchmark the biological relevance of DP, we evaluated its ability to prioritize known cancer- and GC-related genes using multiple reference gene sets [45, 46]. Across all disease progression processes, cancer genes consistently exhibited significantly higher DP values than noncancer genes at all tested hub-degree thresholds ≥35 (Fig. 3B and Supplementary Fig. S3). This trend remained evident even when the analysis was restricted to highly connected hub nodes (degree ≥ 140), where cancer genes still displayed markedly elevated DP values compared with noncancer genes (Fig. 3C).

**Figure 3:**
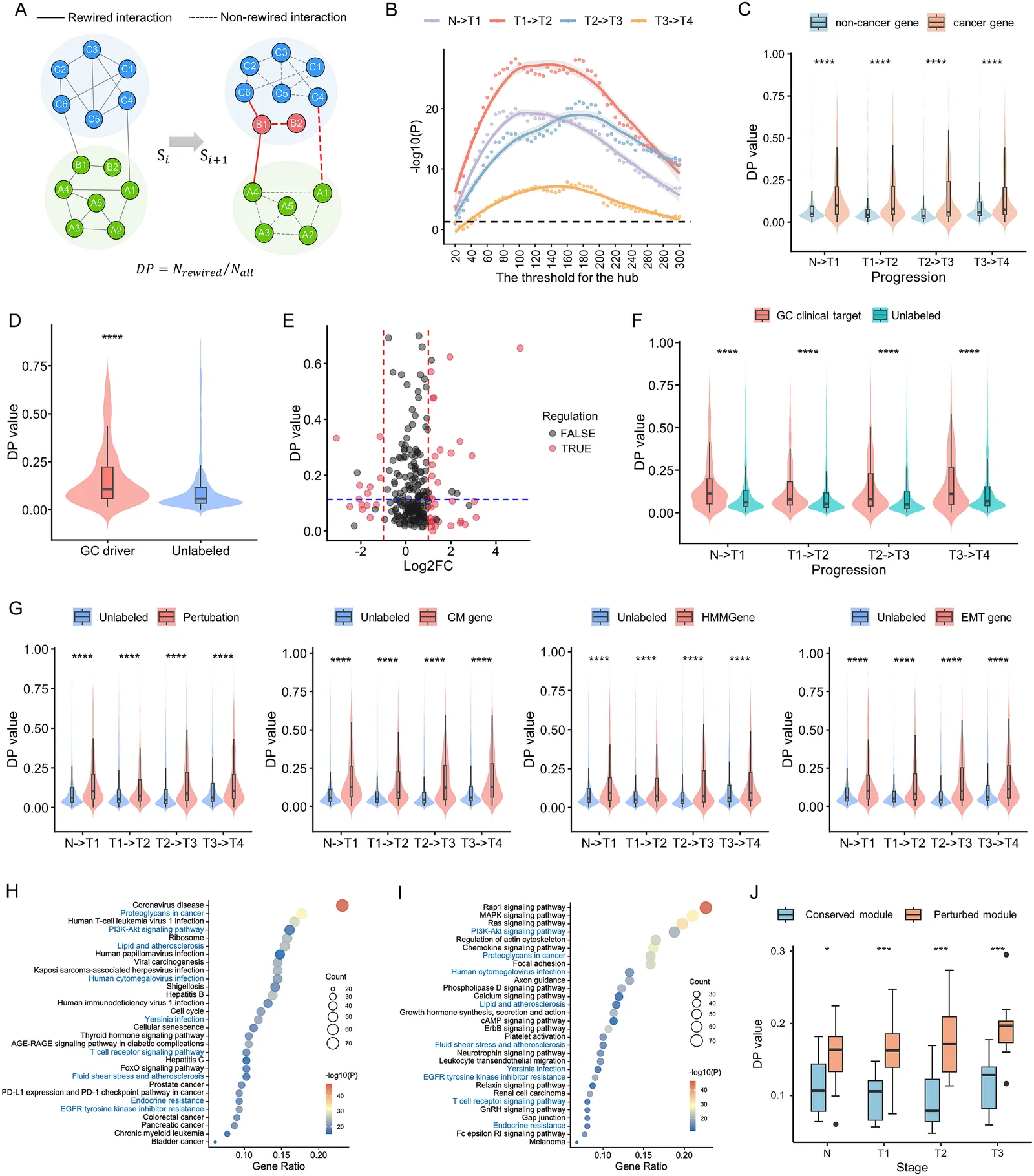
The biological significance of DP. (A) Schematic illustration of the DP metric. (B and C) Differences in DP values between cancer genes and non-cancer genes across different disease progression processes under various hub-degree thresholds (B) and among hub nodes with degree ≥140 (C). (D) Differences in DP values between GC driver and unlabeled genes during the N → T1 transition. (E) DP values and log_2_FC of GC driver genes, with the blue dashed line indicating the threshold for the top 50% of DP values. (F) Comparison of DP values between clinically validated GC targets and unlabeled genes. (G) Differences in DP values between computational perturbation signature genes or cancer metastasis-related genes and unlabeled genes. Top 30 significantly enriched KEGG pathways for the top 10% hub nodes (H) and hub nodes ranked within the top 10% according to DP values (I). Shared pathways were marked in blue font. (J) Comparison of DP values between conserved and perturbed modules during GC progression. ^*^P < .05; ^**^P < .01; ^***^P < .001; ^****^P < .0001 (Wilcoxon’s rank-sum test).

We next evaluated the ability of DP to identify GC-specific disease-related genes. Both reported GC driver genes and clinically validated GC therapeutic targets exhibited significantly higher DP values than unlabeled genes (Fig. 3D–F and Supplementary Fig. S4). Further comparison with conventional differential expression analysis revealed that only a subset of these GC driver genes and clinical targets could be identified based on differential expression, whereas the majority could be prioritized based on their elevated DP values. In addition, perturbation signature genes and cancer metastasis-related genes also exhibited significantly higher DP values than unlabeled genes (Fig. 3G). These results indicate that genes undergoing substantial interaction rewiring are more likely to be involved in tumorigenesis and disease progression, suggesting that DP effectively prioritizes disease-relevant network perturbations beyond simple network connectivity or gene expression changes alone.

We next investigated whether DP identifies functionally distinct biological processes compared with conventional topology-based metrics. Pathway enrichment analysis revealed that the top 10% hub genes selected solely based on network connectivity were predominantly involved in housekeeping functions, including ribosome, cell cycle, and oxidative phosphorylation pathways (Fig. 3H). In contrast, hub genes ranked within the top 10% of DP values were significantly enriched in pathways directly implicated in tumor progression, including Ras signaling, mitogen-activated protein kinase (MAPK) signaling, phosphatidylinositol 3' -kinase (PI3K)–Akt signaling, focal adhesion, regulation of actin cytoskeleton, and epidermal growth factor receptor (EGFR) tyrosine kinase inhibitor resistance pathways (Fig. 3I). These findings demonstrate that DP preferentially identifies dynamic regulatory components associated with malignant progression, whereas conventional hub analysis primarily captures structurally important but biologically ubiquitous network elements.

We further examined the relationship between DP and module-level network reorganization. Across all stage transitions, perturbed modules consistently exhibited significantly higher DP values than conserved modules (Fig. 3J). Moreover, DP values of perturbed modules progressively increased during disease progression, indicating an accumulation of remodeling events. Notably, modules classified as conserved by node composition frequently showed substantial interaction-level rewiring, whereas highly perturbed modules exhibited extensive rewiring despite retaining most of their member genes. These observations suggest that module conservation reflects compositional stability, whereas DP captures interaction-level plasticity that is not evident from module composition alone. Together, these results highlight DP as a complementary metric for characterizing network dynamics during disease progression.

To systematically assess the robustness and performance of DPA, we performed a series of complementary analyses, encompassing resampling, clinical covariate adjustment, random-network testing, and independent benchmark evaluation (Supplementary Results; Supplementary Figs S5 and S6). The results confirmed the reproducibility and statistical significance of the identified perturbation patterns and demonstrated the ability of DP to prioritize disease-relevant genes beyond conventional approaches.

Collectively, DP demonstrated certain biological relevance from disease, functional, and network perspectives. Unlike traditional network metrics that primarily quantify static topological importance, DP directly measures modular rewiring and preferentially highlights genes and modules associated with malignant progression. These results establish DP as a biologically meaningful indicator of dynamic network perturbation and provide the foundation for subsequent identification of key rewiring modules, driver genes, and therapeutic vulnerabilities during GC progression.

### Persistent pertubation modules characterize GC stage progression

Having established the biological relevance of DP, we next sought to identify network modules undergoing substantial rewiring during GC progression. We first compared module prioritization based on DP with conventional conservation analysis using node-composition similarity (*K* value). Notably, the two approaches yielded markedly different rankings (Fig. 4A). For example, modules M21 and M23 in the normal stage exhibited the lowest conservation scores (*K* = 0.216 and 0.213, respectively), suggesting extensive progression divergence. However, DP analysis indicated only moderate perturbation because these modules largely retained their internal interaction architecture. In contrast, module M6 showed relatively high conservation (*K* = 0.64) yet displayed the highest DP value, indicating extensive rewiring despite substantial preservation of member genes. Similar patterns were observed across multiple disease transitions. These findings demonstrate that module conservation based solely on node composition cannot adequately capture interaction-level reorganization, whereas DP directly quantifies structural remodeling arising from modular rewiring.

**Figure 4:**
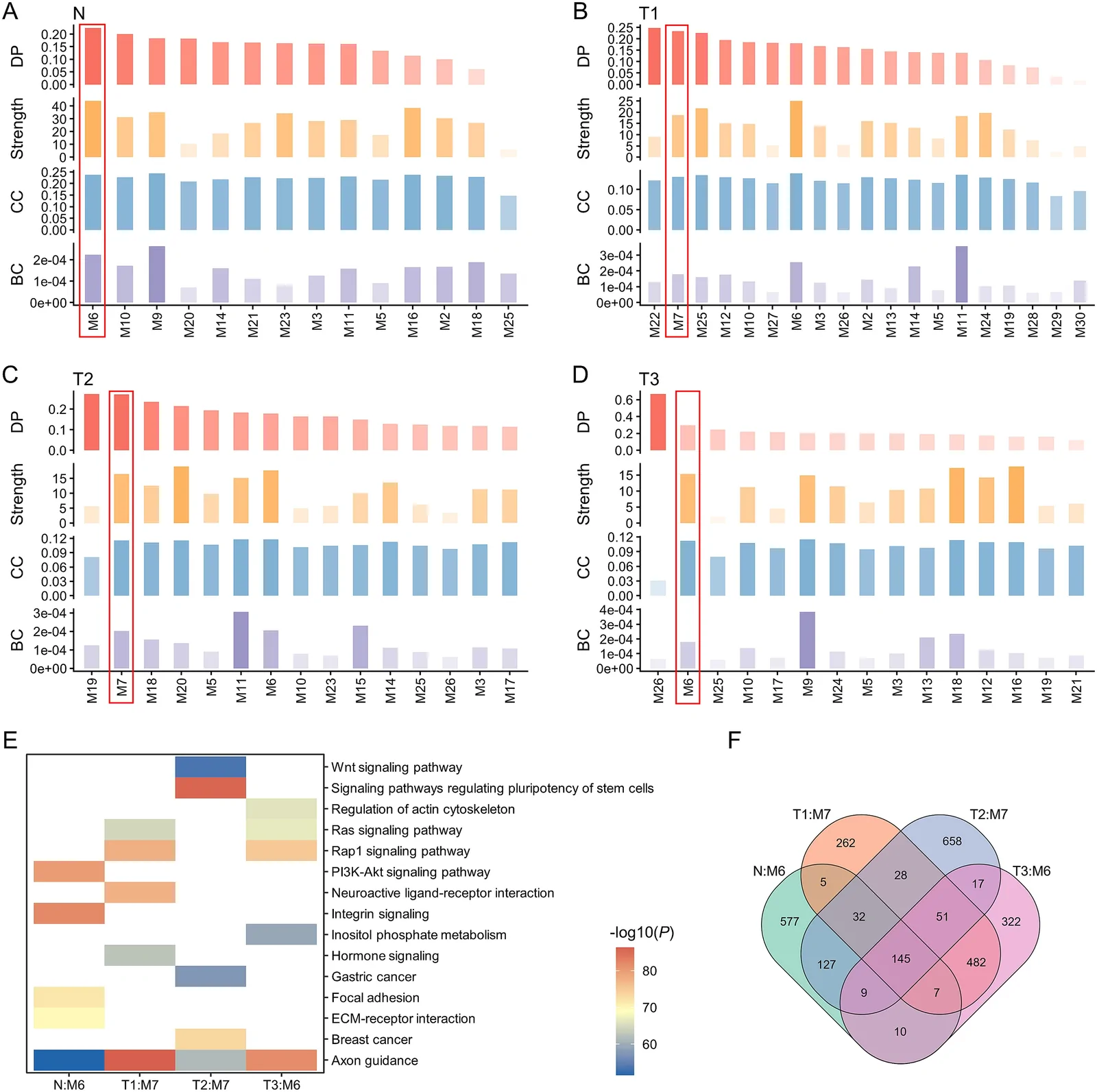
Identification and functional characterization of highly perturbed modules during GC progression. (A–D) Integrative analysis of DP and network topological parameters, including strength, CC, and BC, for modules in the previous stage across the four progression processes. (E) Pathway enrichment analysis of highly perturbed modules. (F) Venn diagram showing the overlap of genes among these four modules.

To identify the most influential rewiring centers during disease progression, we integrated DP with three weighted topological metrics, including strength, betweenness centrality (BC), and closeness centrality (CC). Modules exhibiting both strong perturbation and dominant network positions were considered DPMs. Across successive disease stages, four modules consistently satisfied these criteria, namely M6 (N), M7 (T1), M7 (T2), and M6 (T3) (Fig. 4A–D, Supplementary Table S2). These modules ranked among the top two in DP at their respective stages (DP values: 0.224, 0.233, 0.271, and 0.295) and within the top four across all three topological metrics (Strength: 44.112, 18.748, 16.467, and 15.283; CC: 0.238, 0.131, 0.115, and 0.112; BC: 2.22e-04, 1.78e-04, 2.01e-04, and 1.78e-04). Their consistently high rankings in both perturbation magnitude and topological influence suggest that these modules serve as major organizational hubs driving network remodeling during GC progression.

Functional characterization of DPMs revealed a striking stage-dependent transition pattern (Fig. 4E). The DPM identified in the normal stage was primarily enriched in extracellular matrix organization, focal adhesion, integrin signaling, and PI3K–Akt signaling pathways, suggesting that extensive microenvironmental remodeling accompanies early tumor initiation. During the T1 stage, the dominant DPM shifted toward pathways associated with signal transduction and intercellular communication, including Rap1 signaling, Ras signaling, and neuroactive ligand–receptor interaction pathways. At T2, rewiring became concentrated in stemness-related programs, including Wnt signaling, pluripotency regulation, and GC pathways, indicating acquisition of proliferative and malignant characteristics. Finally, the T3 DPM was enriched in cytoskeletal remodeling and metabolic adaptation pathways, including regulation of actin cytoskeleton and inositol phosphate metabolism, reflecting enhanced invasive and metastatic potential. Together, these findings delineate a stage-ordered functional cascade, progressing from extracellular matrix remodeling to oncogenic signaling activation, stemness acquisition, and invasive adaptation during GC progression.

To provide cellular context for these stage-specific functional transitions, we examined the activity of DPM-associated genes across major cell types using single-cell RNA-seq data from GC samples (Supplementary Fig. S7). The normal-stage DPM showed high activity predominantly in fibroblasts, consistent with its enrichment in extracellular matrix remodeling. The T1 DPM exhibited relatively high activity in endothelial cells, whereas the T2 DPM showed increased activity in epithelial cells, corresponding to enhanced intercellular signaling and stemness-related programs, respectively. The T3 DPM displayed high activity in multiple immune populations, including T cells, NK cells, and myeloid cells, suggesting that late-stage network perturbations increasingly involve interactions with and adaptation to the tumor microenvironment. Together, these single-cell results provide an additional cellular perspective on the stage-dependent functional transitions identified by DPA and suggest that distinct DPMs are preferentially associated with different cellular compartments during GC progression.

Despite their distinct stage-associated functions and cellular preferences, the four DPMs shared a common set of 145 genes (Fig. 4F, Supplementary Table S3), suggesting the existence of a persistent rewiring core underlying multiple stages of GC progression. Rather than acting in a stage-restricted manner, these genes may continuously participate in network remodeling while dynamically altering their interaction partners across disease states. This observation raises the possibility that GC progression is driven not only by stage-specific molecular programs but also by a conserved group of network components that orchestrate rewiring throughout the disease transition. The dynamic perturbation patterns of these shared genes therefore warrant further investigation to identify potential driver genes and therapeutic targets associated with GC progression.

### Modular rewiring programs revel key regulators of GC progression

To investigate how the persistent rewiring core contributes to disease progression, we characterized the modular rewiring programs of the 145 shared genes across successive GC transitions. Unsupervised clustering based on DP profiles identified four distinct perturbation patterns, with four clusters yielding the highest average silhouette coefficient (Fig. 5A, Supplementary Fig. S8), indicating that genes within the rewiring core participate in network remodeling through different stages. Whereas Class 1 and Class 4 displayed relatively stable perturbation patterns, Class 2 and Class 3 exhibited pronounced dynamic changes, suggesting potential roles as stage-specific drivers of disease progression.

**Figure 5:**
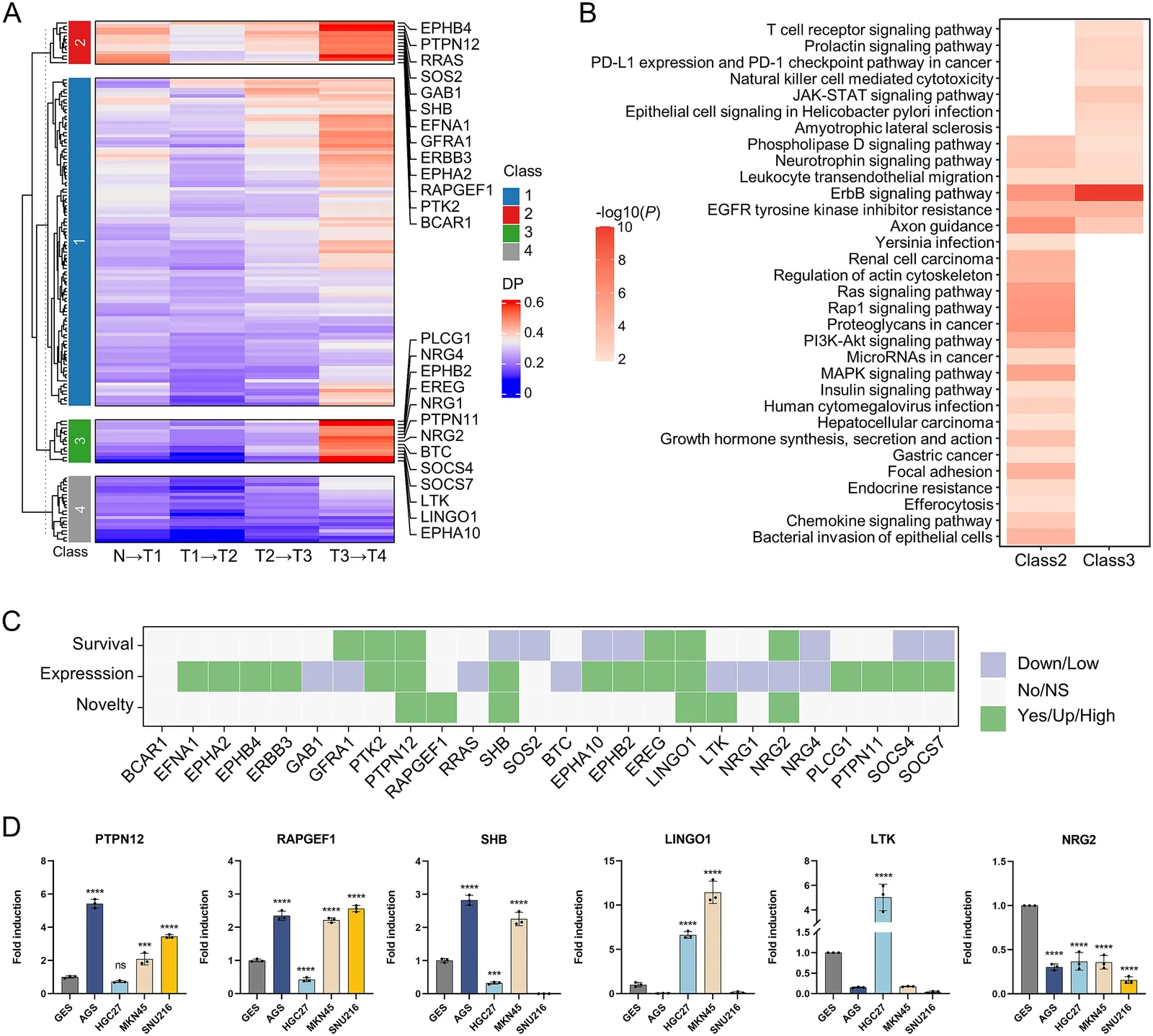
Identification of key gene from highly perturbed modules during GC progression. (A) Clustering of shared genes from modules M6 (N), M7 (T1), M7 (T2), and M6 (T3) based on dynamic changes in DP values across the four GC progression processes. (B) Significantly enriched pathways of Class 2 and Class 3. (C) Integrated analysis of genes in Class 2 and Class 3 based on expression patterns, survival associations, and novelty in GC. Novelty: lacking experimental validation in GC (Yes/No); Expression: direction of dysregulation in GC (Up/Down/NS); Survival: poor prognosis associated with high or low expression, or no significant association (NS). (D) The mRNA-level expression of six novel candidate genes between normal and GC cell lines. ^*^P < .05; ^**^P < .01; ^***^P < .001; ^****^P < .0001.

Genes in Class 2 exhibited a characteristic perturbation pattern marked by strong rewiring during the N → T1 transition, followed by transient attenuation during T1 → T2 and subsequent reactivation throughout later disease stages (Fig. 5A). This transition suggests that these genes may participate in both tumor initiation and subsequent malignant progression. Functional enrichment analysis revealed significant associations with pathways governing signal transduction, cell proliferation, adhesion, migration, and therapeutic resistance (Fig. 5B). These findings indicate that Class 2 genes likely represent a continuously active driver program that orchestrates the establishment and maintenance of malignant phenotypes during GC progression.

In contrast, genes in Class 3 remained relatively quiescent throughout the early and intermediate stages of disease progression but underwent a sharp increase in perturbation during the T3 → T4 transition (Fig. 5A). Functional enrichment analysis showed that these genes were predominantly involved in cytokine signaling, immune regulation, and inflammatory response pathways (Fig. 5B). Unlike Class 2, which reflects persistent oncogenic activity, Class 3 appears to represent a late-stage adaptive program that becomes activated during advanced disease. The abrupt rewiring of immune- and inflammation-related interactions suggests that remodeling of the tumor microenvironment may constitute a critical event driving the final stages of malignant evolution.

Multidimensional analyses demonstrated that these two clusters were not only closely associated with GC progression (Supplementary Table S4), but also enabled the identification of six high-priority candidate genes, protein tyrosine phosphatase non-receptor type 12 (PTPN12), Rap guanine nucleotide exchange factor 1 (RAPGEF1), SH2 domain containing adaptor protein B (SHB), leucine rich repeat and Ig domain containing 1 (LINGO1), leukocyte receptor tyrosine kinase (LTK), and neuregulin 2 (NRG2), that have not yet been experimentally validated in GC (Fig. 5C). Independent reverse transcription-quantitative polymerase chain reaction (RT-qPCR) validation demonstrated significant dysregulation of these six candidate genes in GC cell lines (Fig. 5D), with five genes consistently upregulated and *NRG2* showing reduced expression. Among these candidates, PTPN12 and LINGO1 were prioritized for further investigation based on their robust perturbation patterns, potential clinical relevance, and lack of experimental characterization in GC. Their perturbation patterns remained robust to variations in sample size and clinical covariates (Supplementary Fig. S9A–C). PTPN12 maintained relatively high network perturbation throughout GC progression, whereas LINGO1 exhibited pronounced perturbation predominantly during the T3 → T4 transition (*P* < .05) (Supplementary Fig. S9D), despite neither gene showing significant expression differences between adjacent tumor stages (Supplementary Fig. S9E). Single-cell analysis revealed distinct cell-type expression preferences for PTPN12 and LINGO1, predominantly in endothelial cells and pericytes, respectively (Supplementary Fig. S9F–G). In addition, dysregulation of both genes was linked with poorer survival (Supplementary Fig. S9H), which was extra supported by survival and Cox analyses across multiple independent GC cohorts (Supplementary Figs S10–S12 and Supplementary Table S5). Based on these findings, PTPN12 and LINGO1 were selected for functional validation.

### Functional validation of DPA-prioritized rewiring genes

As shown in Fig. 6A, western blot analysis was first performed in GC cell lines with elevated PTPN12 mRNA expression identified by RT-qPCR prescreening. Compared with human gastric epithelial cell line (GES-1), PTPN12 protein levels were markedly increased in the HER2-positive GC cell line (SNU216) and human gastric adenocarcinoma cell line (AGS), consistent with the upregulated mRNA expression detected by RT-qPCR. Since AGS cells exhibited the most pronounced upregulation of both PTPN12 mRNA and protein expression, this cell line was selected for the establishment of a PTPN12 knockdown model. Compared with parental cells (AGS-NC, negative control) and the nontargeting shRNA control group, three specific shRNAs significantly reduced both the mRNA and protein expression levels of PTPN12 (Fig. 6B and C), indicating successful construction of the knockdown model. Among them, shPTPN12-1# and shPTPN12-2# showed relatively higher knockdown efficiency and were selected for subsequent functional assays. Cell Counting Kit-8 (CCK-8) proliferation assays demonstrated that PTPN12 knockdown significantly suppressed the proliferative capacity of AGS cells (Fig. 6D), suggesting a promotive role of PTPN12 in GC cell proliferation. Meanwhile, wound-healing assays showed markedly reduced wound closure rates and significantly decreased cell migration following PTPN12 knockdown (Fig. 6E), indicating that downregulation of PTPN12 effectively inhibited the migratory ability of GC cells. Furthermore, knockdown of PTPN12 significantly impaired the colony-forming ability of AGS cells, as evidenced by reduced colony numbers, smaller colony sizes, and significantly decreased colony formation rates (Fig. 6F). Collectively, these results demonstrate that PTPN12 promotes proliferation, migration, and colony formation in AGS cells, whereas its downregulation markedly suppresses the malignant phenotype of GC cells.

**Figure 6:**
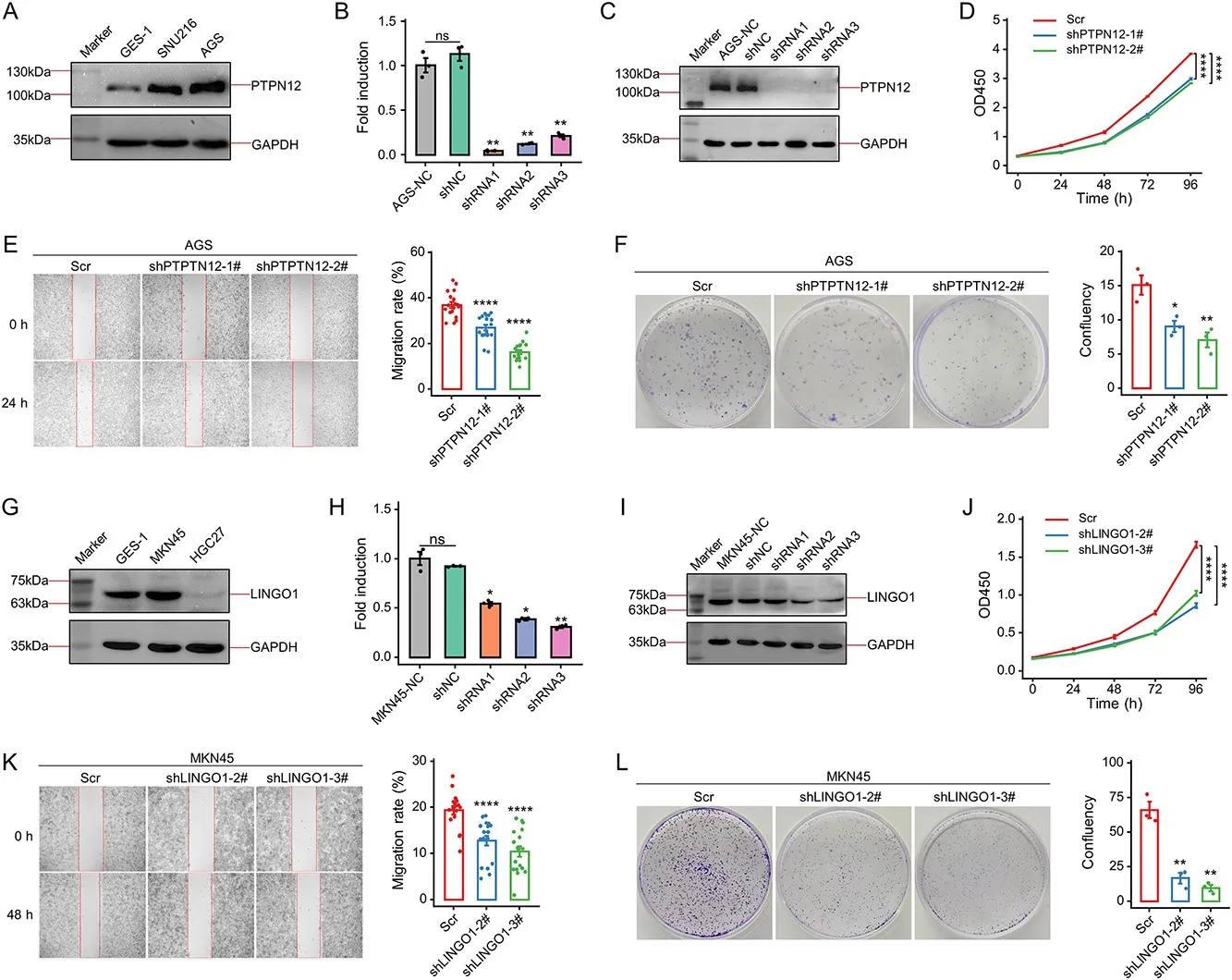
Functional validation of PTPN12 and LINGO1. (A) Western blot validation of PTPN12 protein expression in GC cell lines with high mRNA expression identified by RT-qPCR prescreening. RT-qPCR (B) and WB (C) validation of PTPN12 knockdown cell pools. (D) Cell viability of PTPN12-knockdown AGS cells assessed by CCK-8 assays. (E) Representative wound-healing images and migration rate analysis of AGS cells. (F) Representative images and colony formation rates from colony formation assays of AGS cells. (G) Western blot validation of LINGO1 protein expression in GC cell lines with high mRNA expression identified by RT-qPCR prescreening. RT-qPCR (H) and WB (I) validation of LINGO1 knockdown cell pools. (J) Cell viability of LINGO1-knockdown MKN45 cells assessed by CCK-8 assays. (K) Representative wound-healing images and migration rate analysis of MKN45 cells. (L) Representative images and colony formation rates from colony formation assays of MKN45 cells. ^*^P < .05; ^**^P < .01; ^***^P < .001; ^****^P < .0001.

For LINGO1, western blot analysis was performed in GC cell lines with elevated LINGO1 mRNA expression identified by RT-qPCR prescreening. In contrast to GES-1 cells, LINGO1 mRNA expression was markedly elevated in both HGC27 and MKN45 cells. Nevertheless, LINGO1 protein was barely detectable in HGC27 cells, whereas MKN45 cells exhibited a clear protein band with substantially higher expression than GES-1 cells (Fig. 6G). Therefore, MKN45 cells were selected for subsequent loss-of-function experiments. Transduction with nontargeting shRNA had no obvious effect on LINGO1 expression. Among the three specific shRNAs targeting LINGO1, shLINGO1-2# and shLINGO1-3# achieved efficient knockdown at both the mRNA and protein levels, while shLINGO1-1# displayed only limited inhibitory effects (Fig. 6H and I). Therefore, shLINGO1-2# and shLINGO1-3# were selected for subsequent validations. Functional assays similarly demonstrated that knockdown of LINGO1 significantly impaired the malignant behaviors of MKN45 cells, including reduced cell viability, delayed wound closure, weakened migratory capacity, and decreased colony formation ability (Fig. 6J–L). These findings suggest that LINGO1 exerts tumor-promoting effects in GC cells by facilitating proliferation, migration, and clonogenic growth.

Beyond these functional phenotypes, we next examined transcriptional changes in DPA-prioritized network contexts following PTPN12 or LINGO1 knockdown. RT-qPCR was performed for selected rewiring interaction nodes and downstream transcriptional readouts prioritized by combined score. For the PTPN12-centered network, we selected proline-serine-threonine phosphatase interacting protein 1 (PSTPIP1), paxillin (PXN), SHC adaptor protein 1 (SHC1), BCAR1 scaffold protein, Cas family member (BCAR1), and EGFR as high-confidence network nodes, together with matrix metallopeptidase 2 (MMP2) and BCL2 apoptosis regulator (BCL2) as downstream readouts. For the LINGO1-centered network, nerve growth factor receptor (NGFR), reticulon 4 (RTN4), TNF receptor superfamily member 19 (TNFRSF19), myelin associated glycoprotein (MAG), and reticulon 4 receptor (RTN4R) were selected as network nodes, with Jun proto-oncogene, AP-1 transcription factor subunit (JUN) and Fos proto-oncogene, AP-1 transcription factor subunit (FOS) as downstream readouts. PTPN12 knockdown significantly affected PSTPIP1, PXN, SHC1, EGFR, and BCL2, while LINGO1 knockdown significantly altered the expression of NGFR, RTN4, RTN4R, JUN, and FOS (Supplementary Fig. S13). These results provide additional transcriptional support for the functional relevance of the DPA-prioritized network contexts, although they do not directly validate physical PPI rewiring.

Notably, PTPN12 and LINGO1 were identified solely through their dynamic perturbation characteristics rather than by differential expression alone. Their experimental validation provides independent evidence that modular rewiring captures biologically meaningful GC drivers that may be missed by conventional transcriptomic analyses. Network-level functional enrichment analysis on their rewired interaction partners further revealed distinct functional profiles consistent with their stage-dependent perturbation patterns (Supplementary Results; Supplementary Fig. S14). These findings establish a direct link between dynamic network perturbation and functional tumor-promoting activity, further supporting the utility of DPA for driver gene discovery.

### Modular rewiring-guided drug repurposing reveals therapeutic vulnerabilities in advanced GC

Having identified two major perturbation clusters associated with GC progression, we next explored whether stage-specific rewiring patterns could reveal therapeutic vulnerabilities. Compared with Class 2, which represents a continuously active oncogenic program operating throughout disease progression, Class 3 exhibited a pronounced perturbation surge during the late-stage transition and was predominantly enriched in immune- and microenvironment-related pathways. Because advanced GC is characterized by extensive tumor–microenvironment interactions and remains the primary clinical challenge [47, 48], we hypothesized that the Class 3 signature may capture molecular dependencies particularly relevant to therapeutic intervention in advanced disease.

To systematically identify candidate compounds targeting the Class 3 cluster, we integrated two complementary strategies. First, connectivity map (CMap) was used to identify compounds capable of reversing the Class 3 transcriptional signature. Second, oncoPredict analysis was employed to evaluate the association between Class 3 expression patterns and predicted drug sensitivity across cancer samples. Whereas CMap prioritizes compounds based on transcriptional reversal potential, oncoPredict estimates therapeutic susceptibility from pharmacogenomic profiles. By combining these orthogonal approaches, we aimed to increase the robustness of candidate drug identification (Fig. 7A–C).

**Figure 7:**
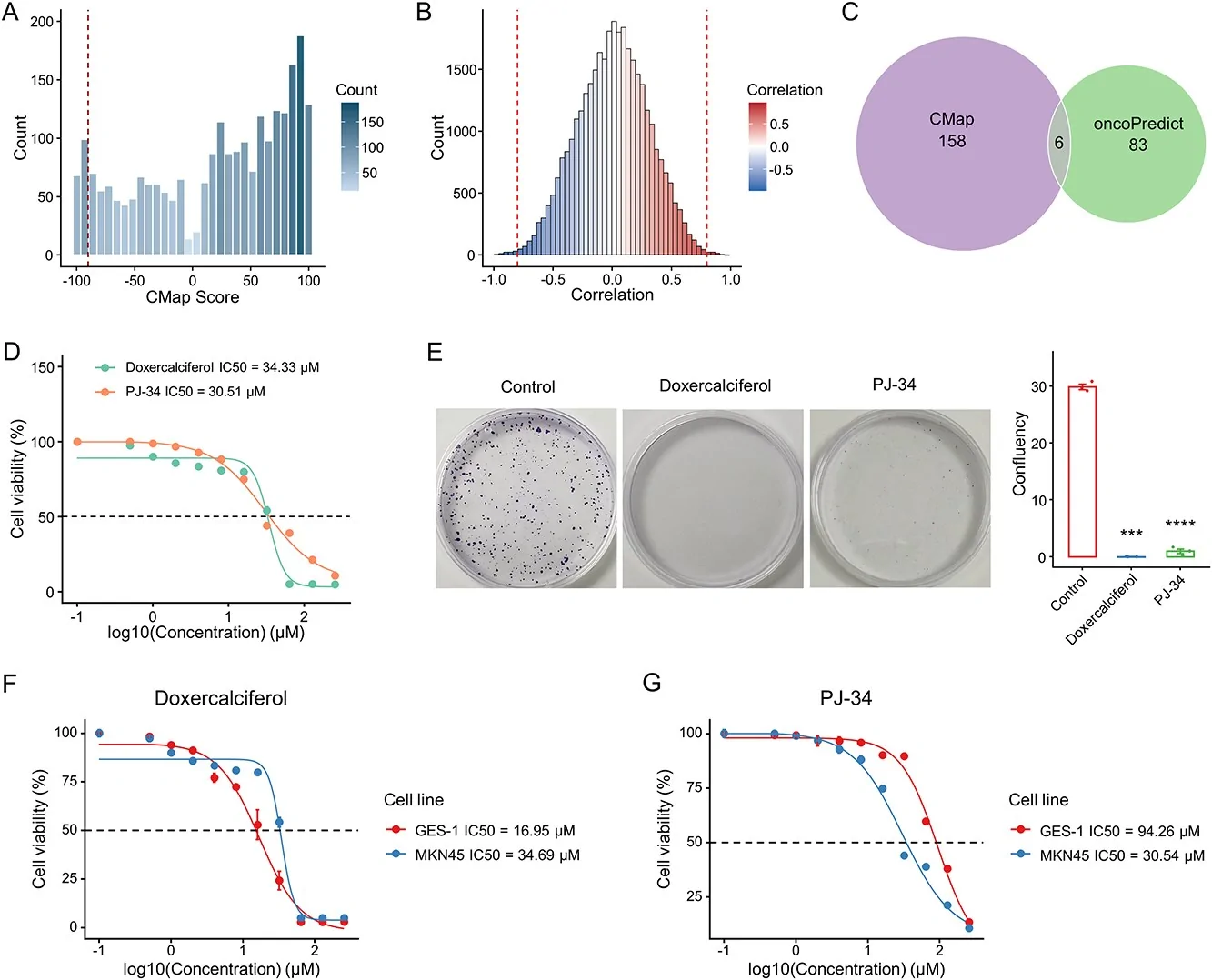
Drug repurposing based on the Class 3 and experimental validation of candidate compounds. (A) Distribution of CMap scores for candidate compounds. The dashed line indicates the selection threshold (CMap score = −90). (B) Distribution of correlations between predicted drug sensitivity and expression profiles. The dashed lines indicate the selection threshold (|correlation coefficient| = 0.8). (C) Overlap of candidate drugs identified by CMap and oncoPredict. (D) Dose–response curves of doxercalciferol and PJ-34 in MKN45 cells determined by CCK-8 assays. (E) Representative images and quantitative analysis of colony formation assays in MKN45 cells following treatment with doxercalciferol or PJ-34. (F) Dose-response curves and selectivity analysis of doxercalciferol in GES-1 and MKN45 cells. (G) Dose-response curves and selectivity analysis of PJ-34 in GES-1 and MKN45 cells.

Application of stringent selection criteria identified 158 compounds from CMap and 83 compounds from oncoPredict analysis. Intersection analysis yielded six high-confidence candidates, including doxercalciferol, PJ-34, cinnarizine, xylazine, lapatinib, and mitomycin-C (Fig. 7C). Notably, lapatinib and mitomycin-C have already demonstrated clinical utility in GC [49], providing indirect support for the validity of the trajectory-guided screening strategy. Among the remaining candidates, doxercalciferol and PJ-34 exhibited the strongest negative associations with the Class 3 signature and were therefore prioritized for experimental validation.

As shown in Fig. 7D, CCK-8 assays showed that both doxercalciferol and PJ-34 significantly inhibited the viability of MKN45 GC cells in a concentration-dependent manner, with IC_50_ values of 34.33 and 30.51 μM, respectively. Consistently, colony formation assays demonstrated that both compounds markedly suppressed the clonogenic capacity of MKN45 cells (Fig. 7E). To further evaluate tumor selectivity, the inhibitory effects of the two compounds were compared between MKN45 cells and normal gastric epithelial GES cells. Doxercalciferol showed greater cytotoxicity toward GES-1 cells (GES-1 IC_50_ = 16.95 μM; MKN45 IC_50_ = 34.69 μM; SI = 0.49), indicating no selective inhibitory advantage against GC cells (Fig. 7F). By comparison, PJ-34 exhibited substantially greater inhibitory activity toward MKN45 cells (GES-1 IC_50_ = 94.27 μM; MKN45 IC_50_ = 30.54 μM; SI = 3.09), demonstrating favorable tumor selectivity. Taken together, although both drugs displayed anti-GC activity, PJ-34 showed a more favorable therapeutic profile owing to its comparable inhibitory efficacy and higher tumor selectivity, making it the more promising candidate for further investigation as a repurposed drug for GC.

## Discussion and conclusion

In this study, we developed DPA, a modular rewiring framework for quantifying interaction-level perturbations across disease states and reconstructing progression-associated programs, and therapeutic vulnerabilities. Conceptually, DPA extends network medicine from the identification of disease modules toward the quantitative modeling of disease-stage transitions. A central finding of this study is that dynamic modular rewiring provides information beyond conventional differential expression analysis. While transcriptional alterations became largely saturated following tumor initiation, extensive perturbations in interaction networks persisted throughout GC progression. This observation suggests that disease evolution is driven not only by changes in gene abundance but also by continuous remodeling of molecular interactions. Importantly, the DP metric does not simply measure edge alterations; rather, it quantifies the extent to which a gene participates in disease-associated network reorganization. From a system biology perspective, DP represents a quantitative measure of biological network plasticity. Recent studies have increasingly recognized network plasticity as a hallmark of cancer evolution, whereby adaptive rewiring of molecular interactions enables transitions between distinct pathological states and therapeutic responses [23, 50]. Our results provide quantitative evidence supporting this concept and demonstrate that interaction-level perturbations can reveal regulatory changes overlooked by conventional transcriptomic analyses.

Compared with existing dynamic network approaches, including differential network analysis, dynamic disease modules, and network states frameworks [51–54], DPA offers several conceptual advantages. First, DPA directly integrates interaction rewiring and network context into a unified perturbation metric, enabling quantitative comparison across sequential disease stages. Second, DPA emphasizes modular reconstruction rather than pairwise network comparison, allowing disease progression to be modeled as a continuous process rather than a collection of independent states. Third, DPA links dynamic perturbations to downstream biological interpretation, including module discovery, driver prioritization, and therapeutic prediction. Together, these features position DPA as a scalable framework for studying disease progression from a network dynamics perspective.

By applying DPA to GC, we identified DPM that collectively delineate a progression stages spanning extracellular matrix remodeling, oncogenic signaling activation, stemness acquisition, and late-stage microenvironmental adaptation. These observations support the emerging view that modules exhibiting persistent rewiring across multiple disease stages may represent regions of enhanced network plasticity that facilitate phenotypic transitions and adaptation [55]. Consistent with this interpretation, perturbation analysis identified two major disease-associated transition programs: a continuously active oncogenic program and a late-stage adaptive program associated with immune and microenvironmental remodeling.

Beyond driver discovery, our results highlight the translational potential of stage-guided therapeutic prioritization. A major challenge in systems medicine is bridging disease phenotypes and therapeutic interventions [56]. By linking dynamic perturbation programs to drug-response prediction and connectivity mapping, DPA translated disease-associated network states into actionable therapeutic hypotheses. The identification of PJ-34 suggests that stage-associated perturbation programs may represent exploitable vulnerabilities in advanced GC. This finding is consistent with the emerging paradigm that network-informed therapeutic strategies should target system-level disease states rather than individual molecular components [57]. Consequently, DPA provides a conceptual bridge connecting disease progression stages, molecular mechanisms, and therapeutic discovery.

Several limitations should be acknowledged. First, DPA was developed using cross-sectional transcriptomic data; therefore, the inferred stage-associated network transitions reflect population-level differences rather than true longitudinal evolutionary trajectories within individual tumors. Accordingly, the identified perturbation patterns should be interpreted as stage-associated network reorganization rather than direct evidence of temporal tumor evolution. Second, the current framework relies on coexpression-derived interaction strengths and does not explicitly model regulatory directionality or causal relationships. Integration of longitudinal cohorts, single-cell multiomics data, and directed regulatory networks in future work may enhance the biological resolution and mechanistic interpretability of DPA. Third, while we selected STRING and the Louvain algorithm to maintain methodological consistency with our prior studies [34, 35], we acknowledge that alternative PPI resources or module detection methods could yield different module partitions. Future evaluations incorporating multiple PPI databases, consensus-based community detection strategies, and systematic validation across additional cancer types and noncancer diseases, as well as comparison with existing network-based methods, would further establish the robustness and broad applicability of the framework [58]. Fourth, although PTPN12 and LINGO1 were functionally validated as drivers in GC cells, the mechanistic link between these genes and the inferred modular rewiring events remains to be directly established. Perturbation experiments coupled with transcriptomic profiling and network reconstruction may help elucidate causal relationships between individual genes and network-level plasticity.

In conclusion, we present DPA as a modular rewiring framework for decoding disease progression and therapeutic vulnerabilities. By quantifying interaction-level perturbations, reconstructing progression stages, and linking network dynamics to driver discovery and drug prioritization, DPA extends network medicine from static disease characterization toward quantitative modeling of disease progression. The proposed framework provides a scalable strategy for uncovering disease mechanisms, identifying therapeutic opportunities, and understanding how biological systems transition between pathological states.

**Key Points**

- Modular rewiring persists despite limited stage-specific transcriptional changes, revealing interaction-level remodeling overlooked by conventional expression analyses.
- DPA introduces a rewiring-based perturbation metric that directly quantifies dynamic network reorganization during disease progression.
- DPA reconstructs disease stages and identifies driver programs as well as experimentally validated regulators associated with GC progression.
- Drug repurposing guided by dynamic perturbation modules identifies PJ-34 as a promising candidate for advanced GC.

## Supplementary Material

Supporting_Information_bbag572(1)

## Data Availability

The GC transcriptomic data used in this study were obtained from the UCSC Xena database (https://xena.ucsc.edu/) with the dataset identifier TCGA–STAD. The human interactome data was downloaded from the STRING database (https://string-db.org/). Drug response and transcriptomic data of cancer cell lines were obtained from the DepMap portal (https://depmap.org/portal/). All datasets analyzed in this study are publicly available from the corresponding databases. The source code is openly available at https://github.com/CSB-SUDA/DPA.
