# Supplementary material for: Dynamic perturbation analysis: a modular rewiring framework for decoding cancer stage progression and identifying therapeutic vulnerabilities
Source: Brief Bioinform. 2026 Oct 19;27(5):bbag572. doi: 10.1093/bib/bbag572 (PMC13677786; doi:10.1093/bib/bbag572)
Supplement: Supplementary file 1 [file supporting_information_bbag572(1).docx]

Dynamic perturbation analysis: a modular rewiring framework for decoding cancer stage progression and identifying therapeutic vulnerabilities

Bin Yang‡^1^, Mi Li‡^2^, Xingyi Liu^1^, Xiaojie Jin*^2^, Guang Hu*^1,3^

^1^Biomedical Basic Research Center (BBRC) of Jiangsu, MOE Key Laboratory of Geriatric Diseases and Immunology, School of Life Sciences, Soochow University, No. 199, Ren'ai Road, Suzhou Industrial Park, Suzhou, Jiangsu, 215123, China

^2^College of Pharmacy, Gansu University of Chinese Medicine, No. 35, Dingxi East Road, Chengguan District, Lanzhou, Gansu, 730000, China

^3^Institute of Health Data Science at Soochow University, No. 199, Ren'ai Road, Suzhou Industrial Park, Suzhou, Jiangsu, 215123, China

‡These authors contributed equally to this work.

Corresponding author. Xiaojie Jin, College of Pharmacy, Gansu University of Chinese Medicine, No. 35, Dingxi East Road, Chengguan District, Lanzhou, Gansu, 730000, China. Email: jinlovedream@163.com; Guang Hu, Biomedical Basic Research Center (BBRC) of Jiangsu, MOE Key Laboratory of Geriatric Diseases and Immunology, School of Life Sciences, Soochow University, No. 199, Ren'ai Road, Suzhou Industrial Park, Suzhou, Jiangsu, 215123, China. E-mail: huguang@suda.edu.cn

# Supplementary Materials and Methods

**Data collection and preprocessing**

### Transcriptomic data

RNA-seq expression profiles, clinicopathological characteristics, and follow-up data of gastric cancer (GC) patients were downloaded from the TCGA-STAD (Stomach Adenocarcinoma) project through the UCSC XENA (https://xena.ucsc.edu/) database. Only samples with both definitive clinical stage information and available RNA-seq data were retained for subsequent analyses. A total of 407 GC samples were initially obtained, of which 384 samples met the inclusion criteria and were included in the final analysis. Based on clinical staging information, the samples were categorized into normal (N), tumor stage I (T1), tumor stage II (T2), tumor stage III (T3), and tumor stage IV (T4) groups, comprising 32, 53, 111, 150, and 38 samples, respectively. The clinicopathological characteristics of GC patients across different tumor stages are summarized in Table S6. Overall, tumor stage was significantly associated with pathological grade, TNM classification, and patient survival status, whereas no significant differences were observed in age, gender, or histological subtype among different stages.

To retain as many biologically relevant genes as possible while filtering out low-quality genes, genes with a maximum FPKM value > 1 and expressed in more than one-third of the samples within each group were retained. Ultimately, 16,411 genes were preserved for downstream analyses.

To evaluate the global transcriptomic features across different stages, the gene expression matrix was subjected to log2 transformation, followed by principal component analysis (PCA) for dimensionality reduction and visualization [1]. Gene dysregulation was evaluated using the Wilcoxon rank-sum test in combination with fold change (FC) in gene expression levels. Genes with adjusted *P* < 0.05 after Benjamini-Hochberg (BH) correction and |log2FC| > 1 were defined as differentially expressed genes (DEGs).

### Cancer-relevant gene benchmarks

To systematically evaluate the ability of DPA to prioritize disease-relevant genes, we constructed multiple reference gene sets representing different biological contexts and levels of supporting evidence, including cancer genes, GC driver genes, clinically validated GC therapeutic targets, computational perturbation signatures, and cancer metastasis-related genes.

First, to establish a stringent cancer benchmark, curated cancer and non-cancer gene sets were constructed following the strategies used in the EMOGI [2] and TREE [3] studies, which contain manually curated lists with higher evidence standards. Known cancer genes were collected from 591 canonical cancer drivers in NCG7.2 and 768 genes in the COSMIC Cancer Gene Census (CGC) [4, 5]. Non-cancer genes were defined as genes considered least likely to be associated with cancer based on available evidence and were compiled using a stepwise exclusion procedure. Starting from the all gene pool, we first excluded genes annotated in NCG. Second, genes included in the Kyoto Encyclopedia of Genes and Genomes (KEGG) “Pathways in cancer” pathway were removed [6]. Third, genes included in cancer-related and oncogenic gene sets in MSigDB were excluded [7]. Finally, genes whose expression was significantly correlated with that of known cancer genes were further removed. The final benchmark therefore comprised 776 cancer genes and 6,445 conservatively defined non-cancer genes.

To further evaluate the ability of DPA to prioritize GC-relevant genes, we separately constructed GC driver-gene and clinically validated therapeutic-target benchmarks. Established GC driver genes were collected from IntOGen (74 genes) [8], NCG (104 genes), and COSMIC, including the CGC (29 genes) and TIER 1 genes from the Cancer Mutation Census (185 genes). Candidate GC driver genes were additionally collected from NCG (62 genes), DriverDBv5 (14,540 genes) [9], and non-TIER 1 genes from the COSMIC Cancer Mutation Census (2,699 genes). After merging the different sources and removing duplicates, 244 established GC driver genes were obtained. To avoid incorrectly treating genes with potential evidence of GC involvement as negative genes, both established and candidate GC driver genes were excluded from the complete candidate gene set, and the remaining genes were designated as unlabeled genes. The resulting benchmark contained 244 established GC driver genes and 4,153 unlabeled genes.

Clinically validated GC therapeutic targets were collected from TTD (728 genes) [10], Open Targets (343 genes) [11], CTD (12 genes) [12], and TargetPair (178 genes) [13], while candidate therapeutic targets were obtained from TTD (645 genes) and Open Targets (29 genes). After merging the sources and removing duplicates, 836 clinically validated GC targets were obtained. Similarly, both clinically validated and candidate targets were excluded from the complete candidate gene set, and the remaining genes were designated as unlabeled genes. The resulting benchmark comprised 836 clinically validated GC targets and 14,838 unlabeled genes for subsequent analyses.

In addition, we collected perturbation and cancer metastasis-related gene sets. 3,396 perturbation signature genes were obtained from the MSigDB [7]. Cancer metastasis-related genes were integrated from several specialized resources, including 5,240 genes from CMGene [14] and TMMGdb [15], which curates metastasis mechanism-associated genes, hallmark of metastasis genes and metastasis pathway genes; and 3,633 genes associated with EMT core signatures from EMTome [16].

**Bioinformatics analysis**

### Statistical significance assessment of DP

To assess whether the node-level DP values observed in the real networks were significantly higher than expected under a random background, we constructed empirical null distributions using randomized networks. For each comparison between adjacent disease stages, the networks were randomized while preserving the node degree distribution and the original module labels, and 1,000 independent randomized networks were generated. The same DPA procedure used for the real networks was then applied to each randomized network to calculate node-level DP values under the random background. For node *i* the empirical *P* value was calculated as:

$\text{P}\text{ = }\frac{\text{1+}\sum_{\text{b}\text{=1}}^{\text{B}} \text{I}\left( \text{DP}_{\text{b}}^{\text{ }\text{random}}\text{ ≥}{\text{ }\text{DP}}^{\text{ }\text{observed}} \right)}{\text{B}\text{+1}}$	(1)

where $\text{DP}^{\text{ }\text{observed}}$denotes the DP value of node *i* in the real network, $\text{DP}_{\text{b}}^{\text{ }\text{random}}$ denotes its DP value in the *b*-th randomized network, *B* is the total number of randomizations (here *B*=1000), and *I* is an indicator function that equals 1 when the specified condition is satisfied and 0 otherwise. The addition of 1 to both the numerator and denominator provides a finite-sampling correction and prevents empirical *P* values of zero. A smaller empirical *P* value indicates that the observed DP of a node is greater than expected under the randomized-network background. Nodes with *P* < 0.05 were considered to exhibit statistically significant dynamic perturbation.

### Module conservation analysis

The Otsuka–Ochiai coefficient (*K*) was used to assess the similarity between different modules by quantifying the extent of node overlap [17], which was defined as follows:

$K=\frac{\left| A\cap B \right|}{\sqrt{|A|\times|B|}}$	(2)

where A and B denote the node sets of two distinct modules, and $|A|$ and $|B|$ represent the number of nodes within these modules. By accounting for differences in module size, this coefficient effectively measures the similarity of node composition between modules and can be used for evaluating module conservation and functional relatedness in biological networks. Larger coefficient values indicate a greater degree of shared nodes and potential functional consistency between modules.

For module comparisons between consecutive disease states, the module ($M_{e}$) in the subsequent state exhibiting the highest *K* value with a given module ($M_{g}$) in the preceding state was defined as its major corresponding module. The corresponding maximum *K* value was then used as the conservation score to quantify the extent to which the module ($M_{g}$) was preserved across consecutive disease stages. Moreover, in the context of gastric cancer progression analysis, modules in the N state with an initial *K* value ≥ 0.6 that also retained *K* values ≥ 0.6 in at least two subsequent stage transitions were defined as conserved modules, whereas the remaining modules were classified as perturbed modules.

### Functional enrichment analysis

Kyoto Encyclopedia of Genes and Genomes (KEGG) pathway enrichment analysis were utilized for genes or network modules using the R package clusterProfiler [6, 18]. Hypergeometric tests were used to evaluate whether the input genes were significantly enriched in specific pathways. To account for multiple hypothesis testing, *P* values were adjusted using the Benjamini-Hochberg (BH) method, and those pathways with an adjusted *P* < 0.05 were considered statistically significant.

### Network topology analysis

Three widely used topological parameters, including weighted degree (strength, S), weighted closeness centrality (CC), and weighted betweenness centrality (BC), were calculated to evaluate the importance of nodes and modules in weighted PPI networks [19]. Strength was defined as the sum of the weights of all edges connected to a node and reflects its interaction capacity within the network.

$S_{i}=\sum_{j}^{N} w_{ij}$	(3)

where *i* is a target node, *j* represents all other nodes, *N* is the total number of nodes, *w* is the weighted adjacency matrix, in which $w_{ij}$ the weight of the tie between *i* and *j*.

CC and BC both rely on shortest-path calculations. Because the interaction weights represent association strength, with larger weights indicating stronger associations, the similarity-based interaction weights were transformed into distances using their reciprocals. Accordingly, the weighted shortest-path distance between two nodes was defined as:

$\text{d}\left( \text{i}\text{, }\text{j} \right)\text{=}\text{min}_{P_{\text{t}}\text{∈}P_{\text{ij}}}\left( \sum_{\left( \text{a}\text{, }\text{b} \right)\text{∈}\text{Pt}} \text{ }\frac{\text{1}}{\text{w}_{\text{ab}}} \right)\text{ }$	(4)

where $P_{\text{ij}}$ denotes the set of all paths connecting nodes *i* and *j*, $\text{Pt}$ represents a path in $P_{\text{ij}}$, and $\text{w}_{\text{ab}}$ represents the interaction weight between two adjacent nodes *a* and *b* along the path. Thus, stronger interactions correspond to shorter edge distances. Then, CC quantifies the average weighted shortest-path distance from a node to all other nodes, and is defined as the inverse total length, reflecting its overall accessibility within the network .

${CC}_{i}=\frac{1}{\sum_{j\neq i}^{N} d\left( i, j \right)}$	(5)

BC measures the frequency with which a node lies on the shortest paths between other nodes and indicates its role in mediating information flow.

${BC}_{i}=\sum_{j\neq i\neq k}^{N} \frac{g_{jk}\left( i \right)}{g_{jk}}$	(6)

where $g_{jk}$ is the number of weighted shortest paths between any two nodes, and $g_{jk}\left( i \right)$ is the number of those paths that go through node *i*.

Together, these metrics characterize node importance from the perspectives of local connectivity, information-flow control, and global network centrality. For module-level analysis, the corresponding topological parameter was calculated as the average value of all member nodes within the module. Dynamic perturbation module (DPM) identification was designed to prioritize modules exhibiting both strong perturbation and prominent topological positions. Specifically, a module was defined as a DPM if it ranked within the top four for each of the four metrics (DP, Strength, BC, and CC).

### Single-cell transcriptomic analysis

The GC single-cell transcriptomic datasets GSE134520 [20] and GSE206785 [21] were obtained from the Gene Expression Omnibus (GEO) database, comprising 29 tumor and 11 normal samples, and 24 tumor and 24 normal samples, respectively. For GSE134520, the data were processed using Seurat. Gene expression data were first normalized using the LogNormalize method, followed by the identification of highly variable genes. The data were then scaled while regressing out the percentage of mitochondrial gene expression. Batch effects across samples were corrected using Harmony, and cell clustering was performed at a resolution of 1 using the first 30 principal components (PCs). Cell clusters were annotated according to canonical marker genes reported in the literature, including B cells (CD79A, MS4A1, and CD79B), epithelial cells (EPCAM, KRT19, KRT8, and CDH1), endothelial cells (PECAM1, VWF, and CDH5), fibroblasts (DCN and LUM), macrophages (CD14, CD163, CD68, and CSF1R), monocytes (LYZ, FCN1, S100A9, and S100A8), mast cells (KIT, TPSB2, and SLC18A2), NK cells (NKG7, KLRD1, and GNLY), T cells (CD3D and CD3E), plasma cells (IGHA1, JCHAIN, and IGKC), and pericytes (PDGFRB, RGS5, and MCAM). For GSE206785, the cell-type annotations provided by the original study were directly used without additional clustering or annotation. Finally, the AddModuleScore function in Seurat was used to calculate the activity scores of each DPM across individual cells to characterize their cell-type-specific activity patterns.

### Clustering analysis

To investigate the perturbation response patterns of shared genes during disease progression, hierarchical clustering analysis was performed based on their DP values across different progression processes [22]. Euclidean distance was first used to measure the similarity of perturbation patterns between genes, followed by complete linkage clustering to construct a hierarchical dendrogram. To determine the optimal number of clusters, clustering solutions with k ranging from 2 to 20 were evaluated using the average silhouette coefficient, and the value of k yielding the maximum average silhouette coefficient was selected as the optimal number of clusters. Based on the optimal clustering solution, shared genes were grouped into distinct clusters to identify gene sets exhibiting similar dynamic perturbation patterns.

### Clinical relevance analysis

To evaluate the clinical relevance of the candidate genes, multiple independent GC cohorts with available survival follow-up data were collected, including GSE84437, GSE62254, GSE15459, GSE15460, GSE57303, GSE34942, GSE26901, GSE13861, GSE28541, and GSE26253 from the GEO database [23]. The association between candidate gene expression and patient clinical outcomes were evaluated using the R packages survival and survminer [24, 25]. For survival analysis, patients were stratified into high- and low-expression groups using an optimal cutoff, the mean, or the median, as appropriate for each cohort. The optimal cutoff was determined using the surv_cutpoint() function by selecting the expression threshold that maximized the survival difference between the two groups. Kaplan-Meier (KM) survival curves were generated, and survival differences between groups were assessed using the log-rank test. To account for multiple testing across candidate genes , *P* values were adjusted using the BH procedure. The *P* < 0.05 was considered significant, indicating an association between gene expression and overall survival.

To avoid potential bias associated with data-driven cutoff optimization or prespecified expression thresholds, univariate Cox proportional hazards regression was additionally performed using continuous gene expression values. Hazard ratios (HRs), 95% confidence intervals (CIs), and Wald-test *P* values were reported, with multiple testing controlled using the BH procedure. KM analysis was used only for visualization purposes.

### Drug prediction analysis

The Connectivity Map (CMap) database [26], which compares disease-associated gene expression signatures with compound-induced transcriptional profiles, was used for drug repurposing analysis. In this study, candidate perturbed genes were submitted to the CMap platform to predict possible therapeutic small molecule drugs. CMap scores range from −100 to 100, where negative scores indicate that the compound-induced transcriptional profile is inversely correlated with the disease signature, suggesting its potential to reverse the disease-associated molecular state and exert therapeutic effects. However, positive scores indicate similar transcriptional profiles and are generally considered to have limited therapeutic potential. A CMap score < −90 was used as the threshold for selecting high-confidence candidate compounds.

Drug sensitivity was further predicted using the oncoPredict package [27]. Transcriptomic profiles of Cancer Cell Line Encyclopedia (CCLE) cell lines and drug response data from the PRISM, GDSC, and CTRP databases were downloaded from DepMap as the training dataset [28], comprising 2,488 compounds. Drug activity was represented by the area under the dose–response curve (AUC), with higher AUC values indicating lower drug sensitivity. The expression profiles of the candidate perturbed genes were used as input to predict drug responses in GC samples, and the correlation between predicted drug sensitivity and expression profiles. Correlation coefficients ranged from −1 to 1, where negative values indicate that higher gene expression is associated with increased predicted drug sensitivity, suggesting that the corresponding compound may be more effective in samples with that expression pattern. Candidate drugs with a correlation coefficient < −0.8 and *P* < 0.05 were considered high-confidence compounds.

**Experimental validation**

**Cell culture**

Human gastric cancer cell lines AGS, MKN45, HGC27 and the human gastric epithelial cell line GES-1 were obtained from Wuhan Sunncell Biotechnology Co., Ltd (Wuhan, China; AGS, SNL-103; MKN45, SNL-173; HGC27, SNL-104; GES-1, SNL-304). SNU216 cells were obtained from Procell Life Science & Technology Co., Ltd. (Wuhan, China; Cat. No. CL-0738). All cells were cultured in RPMI-1640 medium (Gibco, Cat. No. C11875500BT) supplemented with 10% fetal bovine serum (FBS; Gibco, Cat. No. 10099-141) at 37°C in a humidified incubator containing 5% CO₂. Cells were passaged using 0.05% trypsin (Gibco, Cat. No. 25300054) when they reached approximately 80% confluence.

**Reverse transcription-quantitative polymerase chain reaction (RT-qPCR)**

Total RNA was extracted using the MolPure® Flash Cell/Tissue Total RNA Kit (YEASEN, Shanghai, China; Cat. No. 19221ES50). Complementary DNA (cDNA) was synthesized from total RNA using the iScript™ cDNA Synthesis Kit (Bio-Rad Laboratories, Hercules, CA, USA; Cat. No. 1708891). RT-qPCR was performed using QK Platinum SYBR Green Master Mix (Applied Biosystems, Thermo Fisher Scientific, Waltham, MA, USA; Cat. No. A57156) on an Applied Biosystems 7500 Real-Time PCR System (Thermo Fisher Scientific, Waltham, MA, USA). All quantitative primers are given in Table S7. The ∆∆CT method was used to calculate relative mRNA expression.

**Western blot**

Cells were harvested and lysed on ice using radioimmunoprecipitation assay (RIPA) buffer supplemented with a protease inhibitor cocktail. Protein concentrations were determined using a bicinchoninic acid (BCA) protein assay. Equal amounts of total protein were separated by 12% sodium dodecyl sulfate-polyacrylamide gel electrophoresis (SDS-PAGE) and subsequently transferred onto polyvinylidene fluoride (PVDF) membranes. The membranes were blocked with 5% skimmed milk for 1 h at room temperature and then incubated overnight at 4°C with primary antibodies against PTPN12 (1:1,000; Proteintech, Cat. No. 83851-6-RR), LINGO1 (1:1,000; Proteintech, Cat. No. 84375-3-RR), and GAPDH (1:4,000; Proteintech, Cat. No. 60004-1-Ig). After washing three times with Tris-buffered saline containing 0.1% Tween-20 (TBST), the membranes incubated with the PTPN12 and LINGO1 antibodies were incubated with an HRP-conjugated Goat Anti-Rabbit IgG secondary antibody at a dilution of 1:5,000 for 1.5 h at room temperature with gentle shaking.

**Lentivirus infection**

For PTPN12 and LINGO1 knockdown, three shRNA sequences targeting different regions of each gene were designed and cloned into the lentiviral vector pLKO.5-puro. Lentiviral particles were packaged and used to infect AGS cells for PTPN12 knockdown and MKN45 cells for LINGO1 knockdown. After infection, stable cell pools were selected using puromycin. The knockdown efficiency of PTPN12 and LINGO1 was verified by western blotting and RT-qPCR. Following validation, two shRNAs with relatively high knockdown efficiency for each gene were selected for subsequent experiments. The shRNA target sequences are listed in Table S8.

**Cell proliferation analysis**

Cell proliferation and viability were assessed using the Cell Counting Kit-8 assay. For the PTPN12 knockdown assay, AGS cells transduced with scramble shRNA served as the negative control, whereas AGS cells transduced with shPTPN12-1# or shPTPN12-2# were used as the PTPN12 knockdown groups. Cells were seeded in 96-well plates at a density of 2 × 10³ cells/well and cultured in RPMI-1640 medium supplemented with 10% fetal bovine serum. At 0, 24, 48, 72, and 96 h, CCK-8 solution was added to each well, and the plates were incubated at 37°C for 2 h. The optical density of each well was measured at 450 nm using a microplate reader.

For the LINGO1 knockdown assay, MKN45 cells transduced with scramble shRNA served as the negative control, whereas MKN45 cells transduced with shLINGO1-2# or shLINGO1-3# were used as the LINGO1 knockdown groups. Cells were seeded in 96-well plates at a density of 1.5 × 10³ cells/well. CCK-8 detection was performed at 0, 24, 48, 72, and 96 h as described above.

To evaluate the effects of candidate drugs on cell viability, GES-1 cells and MKN45 cells were seeded in 96-well plates at a density of 1 × 10⁴ cells/well and incubated overnight. Cells were then treated with candidate drugs at concentrations of 0, 0.5, 1, 2, 4, 8, 16, 32, 64, and 128 μM for 24 h. CCK-8 solution was subsequently added to each well, and the optical density was measured at 450 nm using a microplate reader.

**Wound healing assay**

Cell migration was evaluated using a wound healing assay. Cells were cultured in RPMI-1640 complete medium supplemented with 10% fetal bovine serum until they reached approximately 80% confluence. Cells in the logarithmic growth phase were trypsinized, counted, and seeded into 6-well plates at a density of 1.5 × 10⁶ cells/well for AGS cells and 4 × 10⁶ cells/well for MKN45 cells. After the cells formed a confluent monolayer, linear wounds were generated using a sterile 200 μL pipette tip. Detached cells and debris were removed by gentle washing with PBS, and the cells were then cultured in serum-free basal medium. Images of the wound areas were captured under a microscope immediately after scratching and recorded as 0 h. The same wound fields were imaged again after 24 h for AGS cells and after 48 h for MKN45 cells. Wound closure was then analyzed quantitatively.

**Colony formation assay**

Colony formation assay was performed to evaluate the clonogenic ability of cells. Cells in logarithmic growth were seeded in 35 mm culture dishes at a density of 1.5 × 10³ cells/dish, with three replicate dishes for each group, and cultured in RPMI-1640 complete medium supplemented with 10% fetal bovine serum. The medium was refreshed every 3 days. After 10 days of incubation, colonies were washed with PBS, fixed with 4% paraformaldehyde at room temperature for 30 min, and stained with 0.1% crystal violet for 60 min. The colonies were then imaged and counted. Colony formation efficiency was calculated as follows: colony formation efficiency (%) = number of colonies / number of seeded cells × 100%. To further evaluate the effects of candidate drugs on the proliferative capacity of gastric cancer cells, the lowest concentration of each drug that produced a significant inhibitory effect on cell viability was selected as the working concentration for the colony formation assay.

**Statistical Analysis**

Statistical analyses and graph construction were performed using GraphPad Prism 9.5.0. Data are presented as the mean ± standard deviation. Comparisons between two groups were performed using an unpaired two-tailed Student’s t-test. Comparisons among three or more groups were performed using one-way analysis of variance (ANOVA). When each experimental group was compared with a single control group, Dunnett’s multiple comparisons test was applied.

# Supplementary Results

**Reliability and performance evaluation of DPA**

To systematically evaluate the reliability and performance of DPA, we next assessed the framework from multiple complementary perspectives, including robustness to sampling variation, sensitivity to potential clinical confounders, statistical significance against randomized network backgrounds, and benchmarking against conventional differential expression and static network topological metrics.

To assess the robustness of DPA to sample size and sampling variability, we repeated the complete analysis 1,000 times using downsampling (T1-T4 reduced to 32 samples) and stage-wise bootstrap resampling, and evaluated consistency with the original results at the edge, module, and node levels using edge-weight correlations, adjusted Rand index (ARI), and DP correlations, respectively. In the downsampling analysis, the edge weights of the T2 and T3 networks were relatively more sensitive to changes in sample size, with correlations of approximately 0.6 with those of the original networks, whereas the edge weights of the T1 and T4 networks showed greater stability (Fig. S5A). At the module and node levels, robustness was broadly comparable across stages, with ARI values of approximately 0.5 for module assignments (Fig. S5B) and correlations generally above 0.6 for node-level DP values (Fig. S5C). Bootstrap resampling yielded similar results: edge-weight correlations with the original networks were approximately 0.7-0.8 across stages (Fig. S5D), module-level ARI values were mainly between 0.5 and 0.6 (Fig. S5E), and node-level DP correlations were approximately 0.65 (Fig. S5F). These results indicate that, although variations in sample size and sample composition exerted some influence on network edge weights and module assignments, the node-level perturbation patterns identified by DPA were generally reproducible.

We further assessed the potential effects of clinical covariates, including age, sex, and tumor grade, on the analysis. Within each disease stage, gene expression profiles were adjusted for these covariates by linear regression, with the residualized expression values subsequently used to reconstruct the stage-specific networks and recalculate DP. After adjustment for these covariates, the resulting edge weights remained highly consistent with the original analysis, with correlations of approximately 0.9, while node-level DP correlations were approximately 0.5-0.6 (Fig. S5G-H). These indicate that adjustment for clinical covariates had a relatively limited effect on overall network structure, although some variation was observed in node-level perturbation estimates, suggesting that the major network perturbation patterns identified by DPA were not solely attributable to these clinical factors.

Moreover, empirical null distributions based on 1,000 randomized networks were used to assess the statistical significance of node-level DP. Across all four adjacent disease-stage comparisons, empirical *P* values were strongly concentrated toward lower values (Fig. S5I), with approximately 75% of nodes exhibiting DP values significantly higher than the random-network background at *P* < 0.05 (Fig. S5J).

Finally, to further benchmark the ability of DP to prioritize disease-relevant genes, we compared it with log2FC and three commonly used network topological metrics, including Strength, CC, and BC. Genes were ranked according to each metric, and performance was evaluated by calculating the recall of benchmark genes among the top-ranked genes across different ranking thresholds. For cancer genes and GC-specific disease-related genes, DP achieved recall comparable to that of the three topological metrics, with all network-based metrics generally outperforming log2FC (Fig. S6). For perturbation signature genes and cancer metastasis-related genes, however, DP achieved higher recall than the topological metrics, whereas log2FC showed the lowest recall (Fig. S6). These results indicate that DP performs comparably to conventional network topological metrics in prioritizing established cancer-related genes, while showing a more pronounced advantage in identifying genes associated with network perturbation and cancer metastasis, consistent with its design to capture dynamic interaction reorganization during disease progression.

Collectively, these analyses support the robustness and non-random nature of the perturbation patterns identified by DPA and demonstrate its complementary value in prioritizing disease-relevant genes associated with dynamic modular reorganization.

## Dynamic functional changes in the rewiring partners of PTPN12 and LINGO1

To further explore the functional significance of the key perturbed genes at the interaction-network level, we performed KEGG pathway enrichment analysis of the rewired interaction partners of PTPN12 and LINGO1. The rewired partners of PTPN12 were involved in a broad range of cancer-related processes, including Proteoglycans in cancer, Choline metabolism in cancer, MicroRNAs in cancer, Central carbon metabolism in cancer, PD-L1 expression and PD-1 checkpoint pathway in cancer, and EGFR tyrosine kinase inhibitor resistance. They were also enriched in several major signaling pathways, including MAPK, PI3K-Akt, Ras, Rap1, JAK-STAT, VEGF, and mTOR signaling, as well as processes related to cell adhesion and cytoskeletal organization, such as focal adhesion, adherens junction, tight junction, and regulation of the actin cytoskeleton; enrichment was observed in immune-related pathways, including chemokine signaling, T-cell receptor signaling, natural killer cell-mediated cytotoxicity, and Th17 cell differentiation (Fig. S14A).

In contrast, the rewired interaction partners of LINGO1 showed a more focused functional profile, with enrichment primarily in Axon guidance, IgSF CAM signaling, and Cell adhesion molecule (CAM) interaction (Fig. S14B), suggesting that its network perturbation was mainly accompanied by alterations in interaction environments related to cell guidance and adhesion. Collectively, the rewired interaction partners of PTPN12 and LINGO1 exhibited distinct functional profiles that were consistent with their different stage-dependent DP patterns. PTPN12 showed persistent and broad network perturbations throughout GC progression, whereas the network perturbation of LINGO1 was predominantly concentrated in the late T3→T4 transition. These findings provide additional interaction-level functional evidence supporting the stage-specific network roles of the key perturbed genes identified by DPA.

# Supplementary Figures

**Figure S1** Overview of transcriptomic characteristics and network construction during GC progression. (A) PCA of normal gastric tissues and tumor samples from four different GC stages. (B) Differential expression analysis across the four progression processes (T1 and N, T2 and T1, T3 and T2, and T4 and T3). (C) Schematic workflow for constructing stage-specific weighted PPI networks and identifying network modules based on transcriptomic profiles and the human interactome.

**Figure S2** Functional enrichment analysis. (A) Significantly enriched KEGG pathways of conserved modules. (B) Significantly enriched KEGG pathways of perturbed modules.

**Figure S3** Heatmap of the mean DP ratio between cancer genes and non-cancer genes across different disease transitions and hub degree thresholds.

**Figure S4** GC disease-related gene prioritization by DP. (A) DP values and log2FC of clinically validated GC targets across all progressions, with the blue dashed line indicating the threshold for the top 50% of DP values. (B) Proportions of cancer genes, GC driver genes, and clinically validated GC therapeutic targets among top-ranked genes across different ranking thresholds. At the more stringent top 10% threshold, these disease-related genes showed higher proportions during the early N→T1 and T1→T2 transitions.

**Figure S5** Robustness and statistical evaluation of DPA. (A-C) Down sampling analysis with 1,000 iterations. Correlations of edge weights between the reconstructed and original networks across disease stages (A), ARI of module assignments (B), and correlations of node-level DP values with the original results (C). (D-F) Bootstrap resampling analysis with 1,000 iterations. Correlations of edge weights between the resampled and original networks (D), ARI of module assignments (E), and correlations of node-level DP values with the original results (F). Correlations of edge weights (G) and node-level DP values (H) between networks constructed after adjustment for clinical covariates and the original networks. Empirical significance assessment of node-level DP based on 1,000 randomized networks, showing the distribution of empirical *P* values (I) and their cumulative distribution (J).

**Figure S6** Benchmarking the gene prioritization performance of DP. Recall of benchmark gene sets among top-ranked genes identified by DP, Strength, CC, BC, and Log2FC across different ranking thresholds. Log2FC represents the absolute value , and only genes with value > 1 within each top-ranking threshold were counted.

**Figure S7** Single-cell transcriptomic analysis of GC. Cell-type annotation (A) and DPM scores across different cell types (B) in the GSE134520 dataset. Cell-type annotation (C) and DPM scores across different cell types (D) in the GSE206785 dataset.

**Figure S8** Determination of the optimal number of clusters. Average silhouette coefficients for hierarchical clustering with the number of clusters (k) ranging from 2 to 20.

**Figure S9** DP, expression patterns, and prognostic significance of PTPN12 and LINGO1. (A-C) Average DP patterns of Class 2 and Class 3 genes based on repeated downsampling (A), bootstrap resampling (B), and adjustment for clinical covariates (C). (D) Dynamic DP patterns of PTPN12 and LINGO1 across GC progression, with empirical statistical significance indicated for each transition. (E) Comparison of PTPN12 and LINGO1 expression between adjacent disease stages. (F-G) Single-cell expression patterns of PTPN12 and LINGO1 in the GSE134520 (F) and GSE206785 (G) datasets. (H) Survival analysis of PTPN12 and LINGO1 using optimal expression cutoffs in TCGA cohort. ns, not significant; **P* < 0.05; ***P* < 0.01; ****P* < 0.001.

**Figure S10** Survival analysis of PTPN12 and LINGO1 using optimal expression cutoffs in independent GC cohorts.

**Figure S11** Survival analysis of PTPN12 and LINGO1 using mean expression cutoffs in independent GC cohorts.

**Figure S12** Survival analysis of PTPN12 and LINGO1 using median expression cutoffs in independent GC cohorts.

**Figure S13** RT-qPCR validation of rewiring-associated interaction nodes of PTPN12 and LINGO1 and their downstream transcriptional readouts. (A) Expression levels of PSTPIP1, PXN, SHC1, BCAR1, EGFR, MMP2, and BCL2 in stable PTPN12-knockdown AGS cells (shPTPN12-2#) compared with AGS cells shNC controls. (B) Expression levels of NGFR, RTN4, TNFRSF19, MAG, RTN4R, FOS, and JUN in stable LINGO1-knockdown MKN45 cells (shLINGO1-3#) compared with MKN45 cells shNC controls. Data are presented as mean ± SD (n = 3). ns, not significant; **P* < 0.05; ***P* < 0.01; ****P* < 0.001; *****P* < 0.0001.

**Figure S14** KEGG analysis of rewired interactions. (A) KEGG pathways of PTPN12-interacting genes that were rewired in at least one disease-stage transition. (B) KEGG pathways of LINGO1-interacting genes that remained non-rewired from N→T1 through T2→T3 but were rewired during T3→T4.

# Supplementary Tables

**Table S1** Correspondence relationships of network modules during GC progression.

|  | Module | | | | | K of the previous module in Progress | | | | Number of module nodes | | | | | Number of nodes shared in Progress | | | |  |
| --- | --- | --- | --- | --- | --- | --- | --- | --- | --- | --- | --- | --- | --- | --- | --- | --- | --- | --- | --- |
| Rank | N | T1 | T2 | T3 | T4 | N→T1 | T1→T2 | T2→T3 | T3→T4 | N | T1 | T2 | T3 | T4 | N→T1 | T1→T2 | T2→T3 | T3→T4 | Average K |
| 1 | M17 | M20 | M21 | M20 | M19 | 0.851 | 0.864 | 0.847 | 0.875 | 702 | 578 | 697 | 570 | 748 | 542 | 604 | 536 | 634 | 0.859 |
| 2 | M19 | M21 | M2 | M22 | M21 | 0.862 | 0.845 | 0.885 | 0.818 | 482 | 529 | 570 | 497 | 427 | 435 | 443 | 433 | 371 | 0.852 |
| 3 | M22 | M23 | M24 | M23 | M11 | 0.732 | 0.864 | 0.854 | 0.846 | 948 | 789 | 910 | 988 | 1028 | 633 | 802 | 826 | 835 | 0.824 |
| 4 | M4 | M4 | M4 | M4 | M4 | 0.820 | 0.822 | 0.836 | 0.789 | 534 | 626 | 591 | 597 | 705 | 474 | 462 | 472 | 484 | 0.817 |
| 5 | M12 | M17 | M12 | M11 | M14 | 0.806 | 0.788 | 0.815 | 0.825 | 474 | 472 | 491 | 463 | 441 | 381 | 380 | 382 | 377 | 0.808 |
| 6 | M15 | M18 | M22 | M2 | M15 | 0.810 | 0.821 | 0.772 | 0.808 | 903 | 897 | 822 | 885 | 911 | 729 | 707 | 690 | 733 | 0.803 |
| 7 | M13 | M15 | M13 | M14 | M12 | 0.778 | 0.789 | 0.823 | 0.769 | 1006 | 825 | 799 | 900 | 959 | 709 | 707 | 783 | 755 | 0.790 |
| 8 | M8 | M9 | M9 | M8 | M7 | 0.641 | 0.651 | 0.687 | 0.672 | 758 | 631 | 567 | 728 | 768 | 443 | 427 | 510 | 513 | 0.663 |
| 9 | M7 | M8 | M8 | M7 | M20 | 0.659 | 0.717 | 0.630 | 0.613 | 1199 | 1124 | 1194 | 1279 | 1580 | 765 | 858 | 780 | 844 | 0.655 |
| 10 | M1 | M1 | M1 | M1 | M1 | 0.689 | 0.663 | 0.592 | 0.673 | 925 | 1166 | 1284 | 1376 | 1200 | 716 | 723 | 668 | 709 | 0.654 |
| 11 | M24 | M16 | M16 | M15 | M16 | 0.673 | 0.645 | 0.668 | 0.628 | 918 | 976 | 953 | 914 | 876 | 637 | 603 | 612 | 563 | 0.653 |
| 12 | M3 | M10 | M3 | M3 | M3 | 0.529 | 0.704 | 0.737 | 0.582 | 1884 | 860 | 1514 | 1750 | 1032 | 673 | 1189 | 1339 | 811 | 0.638 |
| 13 | M9 | M11 | M11 | M9 | M10 | 0.557 | 0.729 | 0.532 | 0.658 | 729 | 499 | 815 | 563 | 770 | 336 | 562 | 341 | 493 | 0.619 |
| 14 | M6 | M6 | M6 | M18 | M6 | 0.641 | 0.588 | 0.569 | 0.600 | 912 | 595 | 512 | 1079 | 1145 | 472 | 402 | 564 | 613 | 0.599 |
| 15 | M16 | M13 | M14 | M12 | M23 | 0.659 | 0.465 | 0.647 | 0.321 | 356 | 302 | 166 | 409 | 406 | 216 | 113 | 247 | 122 | 0.523 |
| 16 | M14 | M14 | M15 | M13 | M9 | 0.497 | 0.531 | 0.527 | 0.351 | 234 | 198 | 164 | 148 | 405 | 107 | 104 | 98 | 108 | 0.476 |
| 17 | M11 | M12 | M7 | M18 | M6 | 0.647 | 0.510 | 0.330 | 0.371 | 794 | 774 | 1067 | 1079 | 1145 | 507 | 469 | 305 | 354 | 0.464 |
| 18 | M10 | M7 | M18 | M6 | M20 | 0.489 | 0.604 | 0.382 | 0.356 | 598 | 1012 | 779 | 1043 | 1580 | 380 | 412 | 302 | 346 | 0.458 |
| 19 | M5 | M5 | M5 | M5 | M5 | 0.540 | 0.413 | 0.420 | 0.432 | 504 | 471 | 803 | 208 | 780 | 263 | 263 | 136 | 271 | 0.451 |
| 20 | M2 | M2 | M5 | M1 | M5 | 0.624 | 0.415 | 0.312 | 0.367 | 530 | 519 | 803 | 1376 | 780 | 327 | 271 | 266 | 236 | 0.429 |
| 21 | M18 | M19 | M17 | M10 | M23 | 0.560 | 0.436 | 0.335 | 0.355 | 180 | 218 | 310 | 556 | 406 | 111 | 103 | 106 | 96 | 0.422 |
| 22 | M20 | M26 | M10 | M17 | M13 | 0.227 | 0.401 | 0.333 | 0.375 | 400 | 238 | 429 | 576 | 589 | 70 | 166 | 160 | 182 | 0.334 |
| 23 | M23 | M12 | M18 | M24 | M18 | 0.212 | 0.153 | 0.407 | 0.227 | 84 | 774 | 779 | 104 | 426 | 54 | 39 | 38 | 43 | 0.250 |
| 24 | M21 | M1 | M1 | M6 | M1 | 0.216 | 0.225 | 0.207 | 0.225 | 136 | 1166 | 1284 | 1043 | 1200 | 86 | 94 | 78 | 91 | 0.218 |

**Table S2** DP values and topological metrics of network modules.

| Group | Module | DP | Strength | CC | BC | DP_  rank | Strength_  rank | CC_  rank | BC_  rank |
| --- | --- | --- | --- | --- | --- | --- | --- | --- | --- |
| N | M6 | 0.224 | 44.112 | 0.238 | 2.22e-04 | 1 | 1 | 2 | 2 |
| N | M10 | 0.201 | 31.320 | 0.226 | 1.71e-04 | 2 | 5 | 7 | 4 |
| N | M9 | 0.183 | 35.227 | 0.243 | 2.63e-04 | 3 | 3 | 1 | 1 |
| N | M20 | 0.181 | 10.443 | 0.208 | 7.01e-05 | 4 | 13 | 13 | 14 |
| N | M14 | 0.168 | 18.447 | 0.217 | 1.60e-04 | 5 | 11 | 11 | 7 |
| N | M21 | 0.166 | 26.758 | 0.226 | 1.09e-04 | 6 | 9 | 8 | 11 |
| N | M23 | 0.164 | 34.414 | 0.222 | 7.95e-05 | 7 | 4 | 10 | 13 |
| N | M3 | 0.163 | 28.075 | 0.224 | 1.25e-04 | 8 | 8 | 9 | 10 |
| N | M11 | 0.161 | 29.123 | 0.230 | 1.58e-04 | 9 | 7 | 5 | 8 |
| N | M5 | 0.133 | 17.421 | 0.216 | 9.00e-05 | 10 | 12 | 12 | 12 |
| N | M16 | 0.114 | 38.527 | 0.237 | 1.65e-04 | 11 | 2 | 3 | 6 |
| N | M2 | 0.099 | 30.443 | 0.232 | 1.67e-04 | 12 | 6 | 4 | 5 |
| N | M18 | 0.060 | 26.663 | 0.228 | 1.88e-04 | 13 | 10 | 6 | 3 |
| N | M25 | 0.000 | 6.010 | 0.147 | 1.35e-04 | 14 | 14 | 14 | 9 |
| T1 | M22 | 0.247 | 9.110 | 0.122 | 1.29e-04 | 1 | 13 | 12 | 10 |
| T1 | M7 | 0.233 | 18.748 | 0.131 | 1.78e-04 | 2 | 4 | 4 | 4 |
| T1 | M25 | 0.225 | 21.732 | 0.135 | 1.61e-04 | 3 | 2 | 3 | 6 |
| T1 | M12 | 0.195 | 15.113 | 0.130 | 1.75e-04 | 4 | 8 | 5 | 5 |
| T1 | M10 | 0.185 | 14.661 | 0.127 | 1.33e-04 | 5 | 9 | 9 | 9 |
| T1 | M27 | 0.182 | 5.292 | 0.115 | 6.50e-05 | 6 | 17 | 17 | 16 |
| T1 | M6 | 0.181 | 25.076 | 0.140 | 2.54e-04 | 7 | 1 | 1 | 2 |
| T1 | M3 | 0.167 | 13.706 | 0.122 | 1.24e-04 | 8 | 10 | 13 | 11 |
| T1 | M26 | 0.162 | 5.458 | 0.116 | 6.42e-05 | 9 | 16 | 16 | 18 |
| T1 | M2 | 0.155 | 15.955 | 0.130 | 1.44e-04 | 10 | 6 | 6 | 7 |
| T1 | M13 | 0.144 | 15.268 | 0.127 | 9.08e-05 | 11 | 7 | 8 | 14 |
| T1 | M14 | 0.142 | 12.918 | 0.124 | 2.28e-04 | 12 | 11 | 11 | 3 |
| T1 | M5 | 0.139 | 8.287 | 0.116 | 7.82e-05 | 13 | 14 | 15 | 15 |
| T1 | M11 | 0.138 | 18.280 | 0.136 | 3.57e-04 | 14 | 5 | 2 | 1 |
| T1 | M24 | 0.107 | 19.602 | 0.129 | 1.02e-04 | 15 | 3 | 7 | 13 |
| T1 | M19 | 0.084 | 12.125 | 0.126 | 1.06e-04 | 16 | 12 | 10 | 12 |
| T1 | M28 | 0.075 | 7.413 | 0.117 | 6.24e-05 | 17 | 15 | 14 | 19 |
| T1 | M29 | 0.033 | 2.293 | 0.084 | 6.48e-05 | 18 | 19 | 19 | 17 |
| T1 | M30 | 0.017 | 4.746 | 0.096 | 1.38e-04 | 19 | 18 | 18 | 8 |
| T2 | M19 | 0.273 | 5.511 | 0.081 | 1.25e-04 | 1 | 13 | 15 | 7 |
| T2 | M7 | 0.271 | 16.467 | 0.115 | 2.01e-04 | 2 | 3 | 3 | 4 |
| T2 | M18 | 0.235 | 12.473 | 0.111 | 1.55e-04 | 3 | 6 | 7 | 5 |
| T2 | M20 | 0.214 | 18.903 | 0.115 | 1.36e-04 | 4 | 1 | 4 | 6 |
| T2 | M5 | 0.193 | 9.731 | 0.106 | 9.08e-05 | 5 | 10 | 9 | 11 |
| T2 | M11 | 0.182 | 15.120 | 0.118 | 3.04e-04 | 6 | 4 | 2 | 1 |
| T2 | M6 | 0.178 | 17.589 | 0.118 | 2.05e-04 | 7 | 2 | 1 | 3 |
| T2 | M10 | 0.165 | 4.856 | 0.101 | 7.81e-05 | 8 | 14 | 13 | 13 |
| T2 | M23 | 0.164 | 5.696 | 0.104 | 6.85e-05 | 9 | 12 | 11 | 14 |
| T2 | M15 | 0.149 | 10.016 | 0.106 | 2.29e-04 | 10 | 9 | 10 | 2 |
| T2 | M14 | 0.127 | 13.567 | 0.112 | 1.12e-04 | 11 | 5 | 5 | 9 |
| T2 | M25 | 0.125 | 6.202 | 0.104 | 8.76e-05 | 12 | 11 | 12 | 12 |
| T2 | M26 | 0.118 | 3.402 | 0.097 | 6.09e-05 | 13 | 15 | 14 | 15 |
| T2 | M3 | 0.117 | 11.342 | 0.107 | 1.12e-04 | 14 | 7 | 8 | 8 |
| T2 | M17 | 0.113 | 11.122 | 0.112 | 1.07e-04 | 15 | 8 | 6 | 10 |
| T3 | M26 | 0.667 | 0.221 | 0.031 | 6.17e-05 | 1 | 15 | 15 | 14 |
| T3 | M6 | 0.295 | 15.283 | 0.112 | 1.78e-04 | 2 | 3 | 3 | 4 |
| T3 | M25 | 0.243 | 2.016 | 0.079 | 5.69e-05 | 3 | 14 | 14 | 15 |
| T3 | M10 | 0.219 | 11.151 | 0.108 | 1.37e-04 | 4 | 7 | 6 | 5 |
| T3 | M17 | 0.216 | 4.519 | 0.097 | 7.10e-05 | 5 | 13 | 11 | 12 |
| T3 | M9 | 0.203 | 14.807 | 0.115 | 3.83e-04 | 6 | 4 | 1 | 1 |
| T3 | M24 | 0.201 | 11.410 | 0.107 | 1.14e-04 | 7 | 6 | 7 | 7 |
| T3 | M5 | 0.197 | 6.404 | 0.095 | 7.05e-05 | 8 | 10 | 13 | 13 |
| T3 | M3 | 0.197 | 10.286 | 0.101 | 1.01e-04 | 9 | 9 | 9 | 9 |
| T3 | M13 | 0.187 | 10.781 | 0.097 | 2.10e-04 | 10 | 8 | 10 | 3 |
| T3 | M18 | 0.184 | 17.185 | 0.113 | 2.35e-04 | 11 | 2 | 2 | 2 |
| T3 | M12 | 0.173 | 14.143 | 0.109 | 1.26e-04 | 12 | 5 | 5 | 6 |
| T3 | M16 | 0.162 | 17.608 | 0.109 | 1.05e-04 | 13 | 1 | 4 | 8 |
| T3 | M19 | 0.160 | 5.274 | 0.096 | 7.11e-05 | 14 | 12 | 12 | 11 |
| T3 | M21 | 0.116 | 5.994 | 0.102 | 8.68e-05 | 15 | 11 | 8 | 10 |

**Table S3** Classification of shared genes based on dynamic DP patterns across GC progression.

|  | Gene | Class |  | Gene | Class |  | Gene | Class |
| --- | --- | --- | --- | --- | --- | --- | --- | --- |
| *1* | SEMA3F | 1 | *51* | CRKL | 1 | *100* | SOS2 | 2 |
| *2* | EPHA8 | 1 | *52* | RET | 1 | *101* | RRAS | 2 |
| *3* | NTN1 | 1 | *53* | SEMA4F | 1 | *102* | PTPN12 | 2 |
| *4* | FGF20 | 1 | *54* | PLXNB1 | 1 | *103* | GAB1 | 2 |
| *5* | REM1 | 1 | *55* | PLXNA4 | 1 | *104* | ERBB3 | 2 |
| *6* | EFNB1 | 1 | *56* | NRP2 | 1 | *105* | PTK2 | 2 |
| *7* | SEMA4G | 1 | *57* | GAB2 | 1 | *106* | GFRA1 | 2 |
| *8* | EFNA2 | 1 | *58* | PLXNA2 | 1 | *107* | EPHB4 | 2 |
| *9* | PIK3R2 | 1 | *59* | EFNA3 | 1 | *108* | EPHA2 | 2 |
| *10* | EFNB3 | 1 | *60* | SEMA6C | 1 | *109* | EFNA1 | 2 |
| *11* | KITLG | 1 | *61* | NRAS | 1 | *110* | RAPGEF1 | 2 |
| *12* | SEMA3G | 1 | *62* | PLXNA3 | 1 | *111* | SHB | 2 |
| *13* | FGF23 | 1 | *63* | ABL1 | 1 | *112* | BCAR1 | 2 |
| *14* | SEMA6A | 1 | *64* | FRS3 | 1 | *113* | PLCG1 | 3 |
| *15* | PLXNC1 | 1 | *65* | SRC | 1 | *114* | EREG | 3 |
| *16* | SEMA7A | 1 | *66* | SHC3 | 1 | *115* | LTK | 3 |
| *17* | PIK3R3 | 1 | *67* | IRS2 | 1 | *116* | PTPN11 | 3 |
| *18* | GRB14 | 1 | *68* | SPRY2 | 1 | *117* | LINGO1 | 3 |
| *19* | PIK3CA | 1 | *69* | NEDD9 | 1 | *118* | NRG2 | 3 |
| *20* | FARP2 | 1 | *70* | SDK2 | 1 | *119* | EPHA10 | 3 |
| *21* | SHC2 | 1 | *71* | GIPC1 | 1 | *120* | EPHB2 | 3 |
| *22* | SEMA3C | 1 | *72* | PLXNA1 | 1 | *121* | NRG4 | 3 |
| *23* | SLIT1 | 1 | *73* | MAP2K7 | 1 | *122* | SOCS4 | 3 |
| *24* | IGF1R | 1 | *74* | EPHB1 | 1 | *123* | BTC | 3 |
| *25* | CBLC | 1 | *75* | FGFRL1 | 1 | *124* | NRG1 | 3 |
| *26* | RASA1 | 1 | *76* | GRB10 | 1 | *125* | SOCS7 | 3 |
| *27* | EPHA1 | 1 | *77* | ARTN | 1 | *126* | PSPN | 4 |
| *28* | PTS | 1 | *78* | SOS1 | 1 | *127* | KLB | 4 |
| *29* | EPHA4 | 1 | *79* | SEMA4B | 1 | *128* | DOK5 | 4 |
| *30* | SEMA3D | 1 | *80* | NTF3 | 1 | *129* | GFRA3 | 4 |
| *31* | PIK3CB | 1 | *81* | SHC1 | 1 | *130* | DRAXIN | 4 |
| *32* | FGFR4 | 1 | *82* | UNC5C | 1 | *131* | MEMO1 | 4 |
| *33* | CRK | 1 | *83* | PLXNB2 | 1 | *132* | MST1R | 4 |
| *34* | NRTN | 1 | *84* | EFNA4 | 1 | *133* | EVA1C | 4 |
| *35* | SEMA3E | 1 | *85* | SEMA4D | 1 | *134* | BCR | 4 |
| *36* | INSR | 1 | *86* | ROBO2 | 1 | *135* | EML4 | 4 |
| *37* | IRS1 | 1 | *87* | ROBO1 | 1 | *136* | RTN4 | 4 |
| *38* | SEMA4C | 1 | *88* | SLIT2 | 1 | *137* | MICAL1 | 4 |
| *39* | PLXND1 | 1 | *89* | ERBB2IP | 1 | *138* | MPZL1 | 4 |
| *40* | SEMA6D | 1 | *90* | PIK3R1 | 1 | *139* | SEMA4A | 4 |
| *41* | EPHB3 | 1 | *91* | GFRA2 | 1 | *140* | EPHA7 | 4 |
| *42* | SLIT3 | 1 | *92* | PLXNB3 | 1 | *141* | BCAR3 | 4 |
| *43* | IGDCC3 | 1 | *93* | FRS2 | 1 | *142* | DOK6 | 4 |
| *44* | UNC5B | 1 | *94* | YES1 | 1 | *143* | EPHA6 | 4 |
| *45* | EPHA3 | 1 | *95* | SEMA6B | 1 | *144* | ROBO3 | 4 |
| *46* | FGFR3 | 1 | *96* | NTF4 | 1 | *145* | PTK6 | 4 |
| *47* | NEO1 | 1 | *97* | FRK | 1 |  |  |  |
| *48* | ERBB4 | 1 | *98* | EPHB6 | 1 |  |  |  |
| *49* | DOK4 | 1 | *99* | SEMA3B | 1 |  |  |  |
| *50* | SPRY4 | 1 |  |  |  |  |  |  |

**Table S4** The GC relevance of genes in Class 2 and Class 3.

| Gene | Class | Description related to gastric cancer(GC) | PMID/Link | Novelty |
| --- | --- | --- | --- | --- |
| SOS2 | 2 | In KRAS-amplified gastric cancer models, inhibition of guanine exchange factors SOS1/2 effectively blocks KRAS downstream signaling activation, thereby enhancing sensitivity to MEK inhibition. | 29808010 | No |
| RRAS | 2 | In gastric cancer, RRAS is aberrantly activated by DNA hypomethylation; knockdown of R-RAS expression leads to loss of cell aggregation, cell atrophy, and a reduction in adherent cell number, promoting gastric cancer cell survival. | 15781621 | No |
| PTPN12 | 2 | For stomach adenocarcinoma (STAD), PTPN12 is highly expressed in tumors, and its upregulation correlates with the incidence, suggesting that PTPN12 may serve as a useful diagnostic biomarker for STAD. | 32536826 | Yes |
| GAB1 | 2 | siRNA-mediated silencing of GAB1 significantly inhibits macrophage-induced invasion and migration of gastric cancer cells. | 23644655 | No |
| ERBB3 | 2 | HER3(ERBB3) is highly overexpressed in gastric cancer and is significantly associated with poor prognosis. Knockdown of HER3 inhibits gastric cancer cell proliferation, induces apoptosis, and suppresses cell motility and migration | 28881753; 30555655 | No |
| PTK2 | 2 | PTK2 is highly expressed in gastric cancer tumor tissues, and si-PTK2 inhibits the proliferation, migration, and invasion of gastric cancer cells. | 28231797 | No |
| GFRA1 | 2 | GFRA1 exerts pro-metastatic and anti-apoptotic effects on gastric cancer cells. Inhibition of GFRA1 has little effect on the proliferation and migration of gastric cancer cells, but it suppresses the formation of liver metastatic niches and increases apoptosis. | 36808605 | No |
| EPHB4 | 2 | EPHB4 is highly expressed in gastric cancer tissues and positively correlated with BC005927; knockdown of EPHB4 significantly impairs gastric cancer cell migration and invasion, whereas its overexpression enhances these capacities | 29383777 | No |
| EPHA2 | 2 | EPHA2 is preferentially overexpressed in gastric cancer, and its overexpression promotes the proliferation, migration, and invasion of gastric cancer cells. | 31376289; 15649254 | No |
| EFNA1 | 2 | EFNA1 is preferentially overexpressed in gastric cancer, and EFNA1–EPHA2 signals inhibit cell growth. Ephrin A1(EFNA1) functions as a ligand of EGFR to promote EMT and metastasis in gastric cancer. | 15649254; 39838173 | No |
| RAPGEF1 | 2 | Somatic demethylation of the first intron of RAPGEF1 occurs in approximately 8% of gastric cancers. | 21399874 | Yes |
| SHB | 2 | SHB serves as a candidate biomarker for response to trastuzumab therapy in HER2-positive gastric cancer patients, and its expression level may predict the efficacy of anti-HER2 treatment. Among GC patients receiving trastuzumab treatment, those with high SHB expression exhibit significantly worse overall survival (OS). Conversely, in the untreated cohort, patients with higher SHB expression tend to have longer survival. | 35264144 | Yes |
| BCAR1 | 2 | BCAR1 exerts oncogenic functions in gastric cancer: its overexpression significantly enhances cell proliferation, migration, and invasion, whereas its knockdown markedly suppresses these capacities. | 37928269 | No |
| PLCG1 | 3 | BPTF-regulated PLCG1 upregulation causes intrinsic erlotinib resistance in GC cells. In the TCGA-STAD cohort, PLCG1 is overexpressed in tumor tissues and correlates strongly with BPTF. The BPTF/PLCG1 axis promotes GC cell proliferation in vitro and in vivo. | 37863665 | No |
| EREG | 3 | EREG is overexpressed in gastric cancer cell lines and promotes cell proliferation, migration, invasion, and inhibits apoptosis via activating the ERK/JNK/p38 and PI3K/Akt signaling pathways, while EREG depletion suppresses tumor growth in vivo. | https://dx.doi.org/10.46701/BG.2021022021125 | No |
| LTK | 3 | Three novel mutations in the KMT2A, LTK, and MST1R genes were identified in a patient with second primary gastric cancer who had a history of colon cancer. | 37287033 | Yes |
| PTPN11 | 3 | PTPN11 is significantly overexpressed in gastric cancer tissues, and its expression level is positively correlated with advanced gastric cancer, suggesting that PTPN11 expression may be involved in the progression from early-stage to advanced gastric cancer. Dihydrotanshinone I (DHT) induces necroptosis and G2/M phase arrest in gastric cancer cells by targeting (inhibiting) PTPN11. | 19690960; 39427814 | No |
| LINGO1 | 3 | None | —— | Yes |
| NRG2 | 3 | Upregulation of NRG2 is significantly correlated with poor prognosis in gastric cancer patients, and NRG2, identified as a potential ligand of ERBB2, is associated with poor prognosis in ERBB2-positive GC. | 35116589; 30123134 | Yes |
| EPHA10 | 3 | EphA10 is significantly overexpressed in gastric cancer tissues, where its high expression is closely correlated with lymph node metastasis, advanced TNM stage, and poor survival; moreover, overexpression of EphA10 in gastric cancer cells promotes proliferation and migration while inhibiting apoptosis. | 40603106 | No |
| EPHB2 | 3 | EphB2 expression is significantly elevated in gastric cancer tissues at both the gene and protein levels, with protein upregulation observed in advanced stages (II, III, IV; P = 0.0072). High EphB2 expression correlates with poor overall survival, and its activation in gastric cancer cells enhances malignant properties by reducing adhesion while accelerating migration and invasion. | 32226496 | No |
| NRG4 | 3 | Compared with matched normal tissues, the mRNA expression levels of HER4 (p = 0.0002) and its ligand NRG4 (p = 0.0009) are downregulated in gastric cancer tumor tissues. High NRG4 expression is an independent poor prognostic factor for overall survival in gastric cancer patients. | 24728052; 38151289 | No |
| SOCS4 | 3 | Luciferase reporter assay confirmed that SOCS4 is a direct target gene of miR-106a-3p. In apatinib-resistant gastric cancer cells (SGC-7901-AR), treatment with a miR-106a-3p inhibitor significantly upregulated SOCS4 mRNA and protein expression, reduced the phosphorylation levels of JAK2 and STAT3, and decreased cell viability. Conversely, knockdown of SOCS4 reversed these effects and restored drug resistance. | 31884511 22127425 | No |
| BTC | 3 | BTC, as a ligand of EGFR, participates in EGFR activation-mediated cell proliferation, epidermal growth factor receptor binding, and positive regulation of cell proliferation. High expression of BTC in gastric cancer is associated with poor survival and correlates with resistance to cisplatin. | 31990955; 28399526 | No |
| NRG1 | 3 | NRG1 serves as a potential prognostic and therapeutic biomarker in gastric cancer. It is significantly overexpressed in gastric cancer cells, and knockdown of NRG1 by siRNA reverses the EMT and migration of gastric cancer cells induced by miR-200 inhibition. | 28573357; 34531912 | No |
| SOCS7 | 3 | SOCS7 is a direct target protein of miR-92b-5p. Downregulation of SOCS7 by gastric cancer cell-derived exosomal miR-92b-5p reduces SOCS7-STAT3 interaction, leading to STAT3 hyperphosphorylation, promoting M2 macrophage polarization, and ultimately enhancing gastric cancer cell migration and invasion. | 39566403 | No |

**Table S5** Cox regression analysis of PTPN12 and LINGO1 in TCGA and independent GC cohorts.

| Gene | N | Event | HR | 95% CI lower | 95% CI Upper | P | adj.P | Dataset |
| --- | --- | --- | --- | --- | --- | --- | --- | --- |
| PTPN12 | 378 | 142 | 1.0208 | 1.0007 | 1.0412 | 0.0419 | 0.8716 | TCGA |
| PTPN12 | 192 | 95 | 1.0003 | 1.0001 | 1.0005 | 0.0019 | 0.0927 | GSE15459 |
| PTPN12 | 248 | 122 | 1.0001 | 1.0000 | 1.0002 | 0.0321 | 0.4465 | GSE15460 |
| PTPN12 | 109 | 55 | 2.7969 | 1.5150 | 5.1634 | 0.0010 | 0.0494 | GSE26901 |
| LINGO1 | 378 | 142 | 0.9841 | 0.9327 | 1.0383 | 0.5583 | 0.8716 | TCGA |
| LINGO1 | 433 | 209 | 1.0013 | 1.0002 | 1.0023 | 0.0179 | 0.1807 | GSE84437 |
| LINGO1 | 300 | 152 | 2.3401 | 1.0898 | 5.0247 | 0.0292 | 0.1820 | GSE62254 |
| LINGO1 | 40 | 32 | 1.9956 | 1.1709 | 3.4010 | 0.0111 | 0.0724 | GSE28541 |

**Table S6** Clinicopathological characteristics of GC patients across different tumor stages.

| Characteristic | Level | Stage I | Stage II | Stage III | Stage IV | Overall *P* | II vs I *P* | III vs II *P* | IV vs III *P* |
| --- | --- | --- | --- | --- | --- | --- | --- | --- | --- |
| n |  | 53 | 111 | 150 | 38 |  |  |  |  |
| Age (mean (SD)) |  | 68.76 (11.41) | 64.98 (10.16) | 65.57 (10.33) | 62.49 (11.01) | 0.043 | 0.148 | 0.970 | 0.381 |
| Age_group (%) | <60 | 11 (21.6) | 34 (30.9) | 46 (30.7) | 16 (43.2) | 0.192 | 0.298 | 1.000 | 0.208 |
|  | ≥60 | 40 (78.4) | 76 (69.1) | 104 (69.3) | 21 (56.8) |  |  |  |  |
| Gender (%) | female | 16 (30.2) | 42 (37.8) | 54 (36.0) | 16 (42.1) | 0.676 | 0.433 | 0.861 | 0.612 |
|  | male | 37 (69.8) | 69 (62.2) | 96 (64.0) | 22 (57.9) |  |  |  |  |
| Grade (%) | G1 | 2 (3.9) | 3 (2.8) | 2 (1.4) | 1 (2.8) | 0.002 | 0.004 | 0.306 | 0.174 |
|  | G2 | 31 (60.8) | 38 (34.9) | 41 (27.7) | 15 (41.7) |  |  |  |  |
|  | G3 | 18 (35.3) | 68 (62.4) | 105 (70.9) | 20 (55.6) |  |  |  |  |
| Histology (%) | Intestinal | 13 (24.5) | 17 (15.3) | 31 (20.7) | 12 (31.6) | 0.361 | 0.097 | 0.740 | 0.502 |
|  | Diffuse | 6 (11.3) | 19 (17.1) | 28 (18.7) | 5 (13.2) |  |  |  |  |
|  | Signet ring | 3 (5.7) | 2 (1.8) | 4 (2.7) | 2 (5.3) |  |  |  |  |
|  | Mucinous | 0 (0.0) | 7 (6.3) | 10 (6.7) | 2 (5.3) |  |  |  |  |
|  | Other | 31 (58.5) | 66 (59.5) | 77 (51.3) | 17 (44.7) |  |  |  |  |
| T (%) | T1 | 17 (32.1) | 1 (0.9) | 0 (0.0) | 0 (0.0) | <0.001 | <0.001 | <0.001 | 0.192 |
|  | T2 | 36 (67.9) | 27 (24.3) | 9 (6.0) | 2 (5.3) |  |  |  |  |
|  | T3 | 0 (0.0) | 70 (63.1) | 78 (52.0) | 14 (36.8) |  |  |  |  |
|  | T4 | 0 (0.0) | 13 (11.7) | 63 (42.0) | 22 (57.9) |  |  |  |  |
| N (%) | N0 | 50 (94.3) | 55 (51.4) | 4 (2.7) | 2 (5.3) | <0.001 | <0.001 | <0.001 | 0.113 |
|  | N1 | 3 (5.7) | 41 (38.3) | 34 (23.0) | 11 (28.9) |  |  |  |  |
|  | N2 | 0 (0.0) | 11 (10.3) | 55 (37.2) | 7 (18.4) |  |  |  |  |
|  | N3 | 0 (0.0) | 0 (0.0) | 55 (37.2) | 18 (47.4) |  |  |  |  |
| M (%) | M0 | 47 (100.0) | 107 (100.0) | 143 (100.0) | 14 (36.8) | <0.001 | 1.000 | 1.000 | <0.001 |
|  | M1 | 0 (0.0) | 0 (0.0) | 0 (0.0) | 24 (63.2) |  |  |  |  |
| Status (%) | Alive | 39 (75.0) | 76 (68.5) | 81 (54.0) | 15 (39.5) | 0.001 | 0.504 | 0.026 | 0.156 |
|  | Dead | 13 (25.0) | 35 (31.5) | 69 (46.0) | 23 (60.5) |  |  |  |  |

**Table S7** Sequence of the RT-qPCR primers.

| PTPN12-forward | ACCACAATGGGGAGGACAAC |
| --- | --- |
| PTPN12-reverse | TGGCCCATAGACGCCCTTTA |
| RAPGEF1-forward | ACAAAAACCTGAGCTGGCTG |
| RAPGEF1-reverse | GAAGAGAGGGCTGAGCTGTG |
| SHB-forward | GTATCACGGAGCCATCAGCA |
| SHB-reverse | AAACCCTGGTTGCTCCTCAG |
| LINGO1-forward | CACCACCATGGGCTTCATCT |
| LINGO1-reverse | CGGGGCACATACTCGATCTC |
| LTK-forward | GCTGAATTCTCCGGGCACC |
| LTK-reverse | CACATTGTGTCTGTGTGGGC |
| NRG2-forward | CTCAAGTCAGTGCAGGACCA |
| NRG2- reverse | CTCTCGGGTGCTGTTGGAG |
| PSTPIP1-forward | TCCAGAAGAGCAAGCTGTCG |
| PSTPIP1-reverse | CTGTATACCCGCTCTGCCTC |
| PXN-forward | ATGGACGACCTCGACGC |
| PXN-reverse | CAAGAACACAGGCCGTTTGG |
| SHC1forward | CTTGGGAGCTACATTGCCTGT |
| SHC1-reverse | TGGTGGAGGTGGCATCTGT |
| BCAR1-forward | GACAAGACCAGCAGCATCCA |
| BCAR1-reverse | TCAAACTCCTCCTTCCCCTGT |
| EGFR-forward | AGTCGGGCTCTGGAGGAAAA |
| EGFR-reverse | CCACCTCCTGGATGGTCTTT |
| MMP2-forward | ACAAAGAGTTGGCAGTGCAATA |
| MMP2-reverse | TCTGGGGCAGTCCAAAGAAC |
| BCL2-forward | GGTGAACTGGGGGAGGATTG |
| BCL2-reverse | GTTCCACAAAGGCATCCCAG |
| NGFR-forward | CACCACCGACAACCTCATCC |
| NGFR-reverse | GCAGCTGTTCCACCTCTTGAA |
| RTN4-forward | TCGGGCTCAGTGGATGAGA |
| RTN4-reverse | GGCTGCTCCTTCAAGTCCAT |
| TNFRSF19-forward | GGGACTGCTTGCCAGGATTT |
| TNFRSF19-reverse | ACAGTGCGGTTCGTAAGGAG |
| MAG-forward | GGATTATGATTTCAGCCTCCCG |
| MAG-reverse | TACCAGACACCATGCACCAC |
| RTN4R-forward | GGCAACCTCACACACCTCTT |
| RTN4R-reverse | CGGTTCTGGTGCAGTAGGAG |
| JUN-forward | TGCCGAAAAAGGAAGCTGGA |
| JUN-reverse | TAAGCTGTGCCACCTGTTCC |
| FOS- forward | TGGCGTTGTGAAGACCATGA |
| FOS- reverse | AGTTGGTCTGTCTCCGCTTG |
| GAPDH-forward | AGGTCGGAGTCAACGGATTTG |
| GAPDH-reverse | GGGGTCATTGATGGCAACA |

**Table S8** Target sequences of the shRNAs.

| shPTPN12-1# | CAGCCGAGTTAAATTTGACATT |
| --- | --- |
| shPTPN12-2# | CCACCAGAAGAATCCCAGAAT |
| shPTPN12-3# | GCTTTGCAGGTTATCAGAGAT |
| shLINGO1-1# | CGTTATCCTACTGGACTACAT |
| shLINGO1-2# | CAGCGAGAACAAGATCGTTAT |
| shLINGO1-3# | CGACAATGACCTCGTCTACAT |

# Reference

1.	Ben Salem K, Ben Abdelaziz A. Principal Component Analysis (PCA), Tunis Med 2021;99:383-389.

2.	Schulte-Sasse R, Budach S, Hnisz D et al. Integration of multiomics data with graph convolutional networks to identify new cancer genes and their associated molecular mechanisms, Nature Machine Intelligence 2021;3:513-526.

3.	Su XR, Hu PW, Li DX et al. Interpretable identification of cancer genes across biological networks via transformer-powered graph representation learning, Nature Biomedical Engineering 2025;9:371-389.

4.	Dressler L, Bortolomeazzi M, Keddar MR et al. Comparative assessment of genes driving cancer and somatic evolution in non-cancer tissues: an update of the Network of Cancer Genes (NCG) resource, Genome Biol 2022;23:35.

5.	Tate JG, Bamford S, Jubb HC et al. COSMIC: the Catalogue Of Somatic Mutations In Cancer, Nucleic Acids Res 2019;47:D941-D947.

6.	Kanehisa M, Goto S. KEGG: kyoto encyclopedia of genes and genomes, Nucleic Acids Res 2000;28:27-30.

7.	Liberzon A, Birger C, Thorvaldsdottir H et al. The Molecular Signatures Database (MSigDB) hallmark gene set collection, Cell Syst 2015;1:417-425.

8.	Gundem G, Perez-Llamas C, Jene-Sanz A et al. IntOGen: integration and data mining of multidimensional oncogenomic data, Nat Methods 2010;7:92-93.

9.	Liu CH, Lai YL, Shen PC et al. DriverDBv4: a multi-omics integration database for cancer driver gene research, Nucleic Acids Res 2024;52:D1246-D1252.

10.	Zhou Y, Zhang Y, Zhao D et al. TTD: Therapeutic Target Database describing target druggability information, Nucleic Acids Res 2024;52:D1465-D1477.

11.	Ochoa D, Hercules A, Carmona M et al. Open Targets Platform: supporting systematic drug-target identification and prioritisation, Nucleic Acids Res 2021;49:D1302-D1310.

12.	Davis AP, Wiegers TC, Johnson RJ et al. Comparative Toxicogenomics Database (CTD): update 2023, Nucleic Acids Res 2023;51:D1257-D1262.

13.	Zhou K, Li X, Yang Y et al. TargetPair: A Single Cell-omics Resource of Clinical Trials-derived Therapeutical Target Combinations for Cancer Therapy, J Mol Biol 2025;437:169399.

14.	Liu Y, Li Z, Lu J et al. CMGene: A literature-based database and knowledge resource for cancer metastasis genes, J Genet Genomics 2017;44:277-279.

15.	Liu HC, Ng KL, Mekala VR et al. TMMGdb-Tumor Metastasis Mechanism-associated Gene Database, Current Bioinformatics 2023;18:63-75.

16.	Vasaikar SV, Deshmukh AP, den Hollander P et al. EMTome: a resource for pan-cancer analysis of epithelial-mesenchymal transition genes and signatures, Br J Cancer 2021;124:259-269.

17.	Rao TR, Ghosh SK, Goswami A. Mining user-user communities for a weighted bipartite network using spark GraphFrames and Flink Gelly, Journal of Supercomputing 2021;77:5984-6035.

18.	Wu T, Hu E, Xu S et al. clusterProfiler 4.0: A universal enrichment tool for interpreting omics data, Innovation (Camb) 2021;2:100141.

19.	Opsahl T, Agneessens F, Skvoretz J. Node centrality in weighted networks: Generalizing degree and shortest paths, Social Networks 2010;32:245-251.

20.	Zhang P, Yang M, Zhang Y et al. Dissecting the Single-Cell Transcriptome Network Underlying Gastric Premalignant Lesions and Early Gastric Cancer, Cell Rep 2019;27:1934-1947 e1935.

21.	Kang B, Camps J, Fan B et al. Parallel single-cell and bulk transcriptome analyses reveal key features of the gastric tumor microenvironment, Genome Biol 2022;23:265.

22.	Du KL. Clustering: a neural network approach, Neural Netw 2010;23:89-107.

23.	Barrett T, Wilhite SE, Ledoux P et al. NCBI GEO: archive for functional genomics data sets--update, Nucleic Acids Res 2013;41:D991-995.

24.	Therneau TM. A Package for Survival Analysis in R. <https://CRAN.R-project.org/package=survival>.

25.	Alboukadel Kassambara MK, Przemyslaw Biecek. survminer: Drawing Survival Curves using 'ggplot2'. <https://rpkgs.datanovia.com/survminer/index.html>.

26.	Zhao Y, Chen X, Chen J et al. Decoding Connectivity Map-based drug repurposing for oncotherapy, Brief Bioinform 2023;24.

27.	Maeser D, Gruener RF, Huang RS. oncoPredict: an R package for predicting in vivo or cancer patient drug response and biomarkers from cell line screening data, Brief Bioinform 2021;22.

28.	Arafeh R, Shibue T, Dempster JM et al. The present and future of the Cancer Dependency Map, Nat Rev Cancer 2025;25:59-73.
